# Supporting non-target identification by adding hydrogen deuterium exchange MS/MS capabilities to MetFrag

**DOI:** 10.1007/s00216-019-01885-0

**Published:** 2019-06-17

**Authors:** Christoph Ruttkies, Emma L. Schymanski, Nadine Strehmel, Juliane Hollender, Steffen Neumann, Antony J. Williams, Martin Krauss

**Affiliations:** 1 Department of Stress and Developmental Biology, Leibniz Institute of Plant Biochemistry, Weinberg 3, 06120 Halle, Germany; 2 Luxembourg Centre for Systems Biomedicine (LCSB), University of Luxembourg, 6 avenue du Swing, 4367 Belvaux, Luxembourg; 3 Eawag: Swiss Federal Institute of Aquatic Science and Technology, Überlandstrasse 133, 8600 Dübendorf, Switzerland; 4 Institute of Biogeochemistry and Pollutant Dynamics, ETH Zürich, 8092 Zürich, Switzerland; 5 iDiv - German Centre for Integrative Biodiversity Research (iDiv), Halle-Jena-Leipzig Deutscher, Platz 5e, 04103 Leipzig, Germany; 6 National Centre for Computational Toxicity (NCCT), United States Environmental Protection Agency, Research Triangle Park, NC 27711 USA; 7 Helmholtz Centre for Environmental Research – UFZ, Permoserstr. 15, 04318 Leipzig, Germany

**Keywords:** Compound identification, In silico fragmentation, Hydrogen deuterium exchange, High-resolution mass spectrometry, Structure elucidation, Metabolomics

## Abstract

Liquid chromatography coupled with high-resolution mass spectrometry (LC-HRMS) is increasingly popular for the non-targeted exploration of complex samples, where tandem mass spectrometry (MS/MS) is used to characterize the structure of unknown compounds. However, mass spectra do not always contain sufficient information to unequivocally identify the correct structure. This study investigated how much additional information can be gained using hydrogen deuterium exchange (HDX) experiments. The exchange of “easily exchangeable” hydrogen atoms (connected to heteroatoms), with predominantly [M+D]^+^ ions in positive mode and [M-D]^−^ in negative mode was observed. To enable high-throughput processing, new scoring terms were incorporated into the in silico fragmenter MetFrag. These were initially developed on small datasets and then tested on 762 compounds of environmental interest. Pairs of spectra (normal and deuterated) were found for 593 of these substances (506 positive mode, 155 negative mode spectra). The new scoring terms resulted in 29 additional correct identifications (78 vs 49) for positive mode and an increase in top 10 rankings from 80 to 106 in negative mode. Compounds with dual functionality (polar head group, long apolar tail) exhibited dramatic retention time (RT) shifts of up to several minutes, compared with an average 0.04 min RT shift. For a smaller dataset of 80 metabolites, top 10 rankings improved from 13 to 24 (positive mode, 57 spectra) and from 14 to 31 (negative mode, 63 spectra) when including HDX information. The results of standard measurements were confirmed using targets and tentatively identified surfactant species in an environmental sample collected from the river Danube near Novi Sad (Serbia). The changes to MetFrag have been integrated into the command line version available at http://c-ruttkies.github.io/MetFrag and all resulting spectra and compounds are available in online resources and in the Electronic Supplementary Material (ESM).

**Electronic supplementary material:**

The online version of this article (10.1007/s00216-019-01885-0) contains supplementary material, which is available to authorized users.

**Figure:**
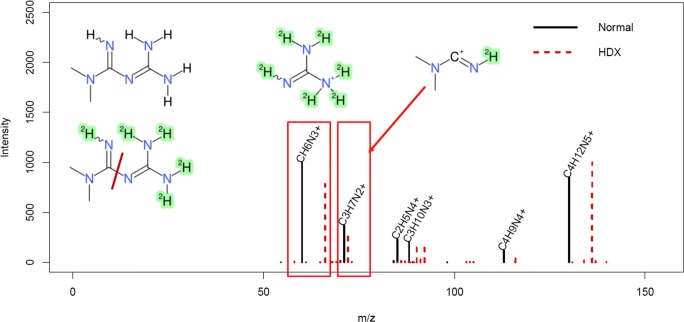
Graphical abstract

## Introduction

The identification of unknown chemicals in complex samples via non-target screening with liquid chromatographic (LC) separation followed by high-resolution(HR) mass spectrometric (MS) analysis remains challenging due to the vast chemical space and still relatively limited coverage of spectra in reference libraries [1, 2]. While techniques such as nuclear magnetic resonance (NMR) spectroscopy yield rich structural information and are well-suited for structure elucidation, NMR is often unachievable with the low concentrations available in complex samples. In LC-HRMS, information about structural properties is obtained by fragmenting detected substances to yield MS/MS spectra. The resulting spectra can then be compared to spectral libraries, or interpreted by software using in silico fragmentation approaches. Unlike NMR, however, the MS/MS spectra typical in LC-HRMS/MS are often information-poor. Thus, alternative ways of obtaining additional structural information are needed for non-target identification methods reliant on LC-HRMS. While techniques such as direct labelling experiments can be used in metabolomics experiments to gain additional information [3, 4], this is impractical in the context of most complex real-world samples, such as environmental samples.

Recently, the inclusion of additional metadata within the in silico fragmenter MetFrag was shown to greatly improve the identification success in the environmental context [5]. While 6% of structures were correctly ranked initially using in silico fragmentation alone with PubChem as a database in this study, this increased to 71% when including metadata such as the retention time, reference, and patent information. Similar results were observed for other in silico fragmenters in the 2016 CASMI contest [6, 7]. However, most metadata scoring terms themselves do not explicitly include the use of structural information to limit candidates, beyond the fragmentation score. While metadata terms such as patent and reference counts provide useful information in some contexts, these could potentially bias the results towards well-known substances and are not useful where no external information is available for the sample or candidate, such as for unknown metabolites or transformation products. Including the retention time alone (without reference information) did not improve candidate ranking greatly [5]. Further approaches for identification, especially in metabolomics, are reviewed elsewhere (e.g., [2]). However, additional ways of obtaining structural information are needed for non-target identification methods reliant on LC-HRMS. One such method of obtaining additional information can be achieved by modifying the analytes prior to performing HRMS, e.g., using hydrogen-deuterium exchange (HDX). This approach is used in proteomics for probing conformation and structural dynamics (with different experimental setups) and has been used occasionally for structure elucidation of small molecules over the last decades (e.g., [8–12]). HDX experiments can be used to provide information about which functional groups may be present in the compound of interest. When the chromatographic system is flooded with deuterated solvents (e.g., D_2_O instead of H_2_O, MeOD instead of MeOH), the “exchangeable hydrogens” can be replaced (i.e., exchanged) with deuteriums. When combined with routine (undeuterated—hereafter termed “normal”) measurements, the changes in the fragmentation pattern can yield information about the substructures in the molecule. While this experimental setup is quite expensive due to the relatively large amounts of deuterated solvents required, cheaper methods such as post-column deuteration tend to yield very complex deuteration patterns due to changing fractions of undeuterated and deuterated solvents along an LC gradient elution that require rigorous statistical analysis [8, 13]. This approach is therefore less useful for the identification of unknown substances at this stage.

**Fig. 1:**
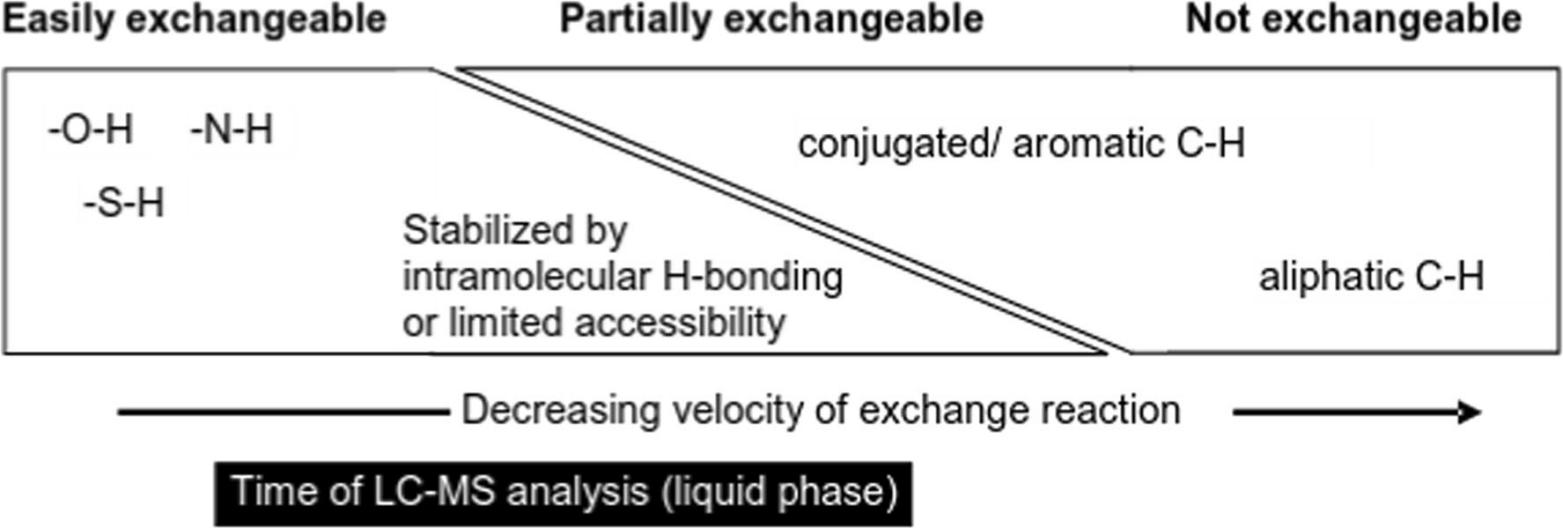
Conceptual view of the degree of exchangeability of hydrogens relative to the timescale of LC-MS analysis

There are essentially three classes of “exchangeable” hydrogens, shown conceptually in Fig. 1, although the borders between the classes are blurred. The “easily exchangeable” hydrogens attached to the heteroatom groups (OH, NH, SH) would generally be completely exchanged in experiments with a deuterium-flooded chromatographic system [14]; typically, the exchange reactions take place in the microsecond to millisecond time range. Those that are sterically hindered or stabilized by hydrogen bonding may take longer to exchange (starting from several millisecond to minutes), but this is still anticipated to occur in most cases within the contact time in the LC system. Partially exchangeable hydrogens, including some conjugated and aromatic hydrogens (e.g., those on pyrrole rings [15] or affected by keto-enol tautomerism [16]), may also exchange in the liquid phase (during LC separation) and/or the gas phase (during ionization and in the MS), with exchange rates depending strongly on experimental conditions [15–17]. However, as shown in Fig. 1, the “unexchangeable” hydrogens, i.e., aliphatic and most aromatic carbons (CH) would not be expected to exchange during an LC-MS run. Thus, a first hypothesis is formed for structure elucidation of small molecules:

All “easily exchangeable” hydrogens should be replaced with deuterium; some conjugated or aromatic hydrogens may be replaced with deuteriums, whereas any aliphatic and most aromatic CH hydrogens would be expected to remain intact.

The influence of deuterium exchange in MS experiments is relevant in both MS1 (full scan) and MS/MS experiments. As deuterium (atomic mass 2.014102 Da) has a different mass to hydrogen (atomic mass 1.007825 Da), the number of deuteriums can be readily determined by the mass difference between the normal and deuterated ion in the full scan (MS1). As the system is flooded with deuterium, the typical ions expected in positive electrospray ionization are no longer [M+H]^+^, but rather [M+D]^+^; thus, the presence of two D in the detected ion indicates one exchangeable hydrogen and one D^+^ adduct, and so on. In negative ESI, the absence of a mass difference indicates one exchangeable hydrogen, which is abstracted by the ionization process to form an [M-D]^−^, with an *m/z* identical to the [M-H]^−^ ion in the undeuterated eluents (note that without an exchangeable H, ionization in negative mode is difficult). From this information, it is possible to determine the maximum number of easily exchangeable hydrogens available on the molecule. The readiness of partially exchangeable hydrogens to be exchanged within the timeframe of the LC method requires further investigation and this was considered throughout this study. Beyond the full scan, the deuterium mass shift will also be reflected in the MS/MS fragments, and the existence of a deuterated fragment in the MS/MS of the deuterated compound can give valuable information about the molecular structure of the compound.

Thus, the aim of this study was to investigate how hydrogen-deuterium exchange experiments could assist structural elucidation in non-targeted HR-MS experiments using high-throughput, automated in silico fragmentation techniques. The in silico fragmenter MetFrag was modified to include additional scoring terms to account for the HDX starting with the theory discussed above and tested on small datasets. Once the method was established, it was evaluated on a set of several mixtures of environmental chemicals containing 762 unique compounds and analyzed in both positive and negative mode, as well as a smaller dataset of 80 metabolites. HDX experiments were then performed on a water sample from the river Danube near Novi Sad (Serbia) to assess the feasibility of applying HDX experiments in the context of a complex real-world water sample.

## Materials and methods

### Experimental data sets

#### Set 1: Deuterated standards and Orbitrap

To ensure that MetFrag accounted for deuterium exchange substitution correctly during the in silico fragmentation, the initial development was performed on stably labeled deuterated substances (typically used as internal standards) where the location of the deuterium atoms (in the precursor) was known. This also served to diagnose any unexpected phenomena in the fragmentation. A mix of internal standards (1 μg/L) was measured on an LTQ Orbitrap XL (Thermo Scientific) with electrospray ionization in positive mode. LC separation was performed in advance on a Kinetex Core-Shell C18 column (3.0 × 100 mm, 2.6 μM particle size) from Phenomenex with H_2_O/MeOH (both with 0.1% formic acid) at a flow rate of 200 μL/min and a gradient of 90/10 at 0 min, 80/20 at 3.2 min, 5/95 at 17.8 min, 5/95 at 37.8 min, 90/10 at 37.9 min, and 90/10 at 47 min. MS/MS scans were obtained using both higher energy collision-induced dissociation (HCD) at nominal collision energy (NCE) of 100 and collision-induced dissociation (CID) at 35 NCE, an MS/MS isolation width of 1.3 *m/z*, and resolution of 15,000. Spectra were extracted for DEET-d7, metolachlor-d6, and carbamazepine-d10, summarized in ESM Table S1.

#### Set 2: HDX and QToF-MS

Individual compounds were dissolved in MeOH/H_2_O 80/20 (*v*/*v*) at a concentration of 10 mM. Then, ten compounds were combined to one synthetic mixture to give 1 mM and the final concentration of each mixture adjusted to 100 μM using MeOH/H_2_O 50/50 (*v*/*v*). Following this, 100 μL was dried down and the residue redissolved in 100 μL acetonitrile/deuterium oxide 50/50 (*v*/*v*), ultrasonicated for 5 min at room temperature, centrifuged at 16,000×*g* for 2 min, and the supernatant injected onto an UPLC-QTOFMS system (Waters, Eschborn, Germany; Bruker Daltonics, Bremen, Germany) with ESI ionization. For the normal (native, undeuterated) samples, water/formic acid, 99.9/0.1 (*v*/*v*), was used as eluent A and acetonitrile/formic acid, 99.9/0.1 (*v*/*v*), as eluent B. In contrast, for the deuterium-exchanged samples, deuterium oxide/formic acid, 99.9/0.1 (*v*/*v*), was applied as eluent A and acetonitrile/formic acid, 99.9/0.1 (*v*/*v*), as eluent B.

Each mixture was measured in both positive and negative ion modes according to [18]. CID mass spectra were acquired using the respective [M+H]^+^, [M-H]^−^, or their deuterated equivalent masses, isolated inside the quadrupole using an isolation width of 3 *m/z* and fragmented inside the collision cell after applying two collision energies (10 eV and 20 eV). All instrument parameters were maintained as previously described in [18]. The resolution was 10,835 (*m/z* 922) in positive mode and 9632 (*m/z* 1034) in negative mode, with a mass accuracy of 5 ppm. The MS and MS/MS data were processed with DataAnalysis 4.2 (Bruker Daltonics, Bremen, Germany) prior to use with MetFrag as previously described [19]. Spectra from kinetin, N-(3-indolylacetyl)-L-valine, o-anisic acid, and phlorizin were used in the results presented further below (see ESM Table S2 for more information).

#### Set 3: Large standard set for HDX and Orbitrap

A total of 22 mixes with 850 substances, already in use at UFZ, were used to measure the large standard set (762 unique substances, i.e., 677, 82, and 3 substances were present once, twice, or three times, respectively, due to the use of the various mixes in the laboratory—see ESM Table S3a). Each mix contained between 10 (mix 15) and 94 (mix 13) substances. Each substance in each mix was assigned a unique identifier, starting at 8000 (a 4-digit number is necessary for RMassBank processing)—such that standards present in more than one mix had two or three identifiers. Each mix was checked for isobars and “near isobars” (substances that would potentially fall within the same MS/MS isolation window of 1.3 *m/z*); the corresponding identifiers were logged for quality control (see ESM Table S3b). For instance, if the presence of an isobar or near isobar could not be excluded, the substance was eliminated from the test set as the spectral quality could not be guaranteed.

The reference standards were purchased from various suppliers at a minimum purity of 97% and spiked in the mixes at a concentration of 1 μg/mL. These mixes were then measured on an LC system coupled to a HR-MS/MS (Q Exactive Plus, Thermo). The Ultimate 3000 LC system (Thermo) used a Kinetex C18 EVO column (2.1 × 50 mm, 2.6 μM particle size), with a 2.1 × 5 mm pre-column from Phenomenex and an injection volume of 5 μL. The gradient was 95/5 at 0 min, 95/5 at 1 min, 0/100 at 13 min, and 0/100 at 24 min at 300 μL/min. For normal measurements, solvents A and B were H_2_O and MeOH, both with 0.1% formic acid. For the deuterated measurements, the solvents were deuterated water (D_2_O, 99.9 atom-% D, Sigma-Aldrich) and deuterated methanol (MeOD, i.e., CH_3_OD, 99.5 atom-% D, Sigma-Aldrich), both containing 0.1% (*v*/*v*) undeuterated formic acid. Electrospray ionization (ESI) in positive and negative mode was used. MS1 was acquired at a nominal resolving power of 70,000 (referenced to *m/z* 200); MS/MS were acquired at R = 35,000 using data-dependent acquisition with 5 MS/MS scans following each full scan MS1 and an inclusion list adjusted to each mix. The pesticide mix (mix 13, containing 94 substances) was run three times in positive mode with different inclusion lists to ensure that MS/MS of all compounds were obtained. Higher energy collision dissociation (HCD) was used with stepped 20/35/50 nominal collision energy units (NCE) and an isolation window of 1.3 *m/z*. All runs were obtained using a range of *m/z* = 100–1000, except for low mass range runs done on the polar compound mix (mix 19), which was between *m/z* = 60 and 600. An overview of the mixes and the original acquisition data are given in ESM Table S3a and b, respectively. In addition to this, the polar compound mix (mix 19) was also re-measured on a Synergi Polar RP column (100 × 3.0 mm, 2.5 μM particle size, Phenomenex). The dataset for CASMI 2016 [6] was formed from the initial normal measurements of these mixes. A full list of substances and further details (structure, predicted ion masses, etc.) are given in ESM Table S3c.

#### Environmental water sample

A well-studied sample from the SOLUTIONS project [20] was used to scope the potential to apply HDX to complex environmental samples. The sample was collected from the river Danube near Novi Sad (Serbia) in the plume of an untreated wastewater inlet using on-site large volume solid-phase extraction and enriched 500-fold for analysis as detailed in [21, 22]. The sample was measured under normal and HDX conditions with a data-dependent top 6 experiment (without an inclusion list) and the same collision energies and other conditions as for the large standard set described above, using the Kinetex column. The target analysis results from [22] were used to direct the data evaluation presented in this manuscript, along with a list of suspect surfactants [23–25].

### Data processing (set 3)

#### HDX prediction and registration

**Fig. 2:**
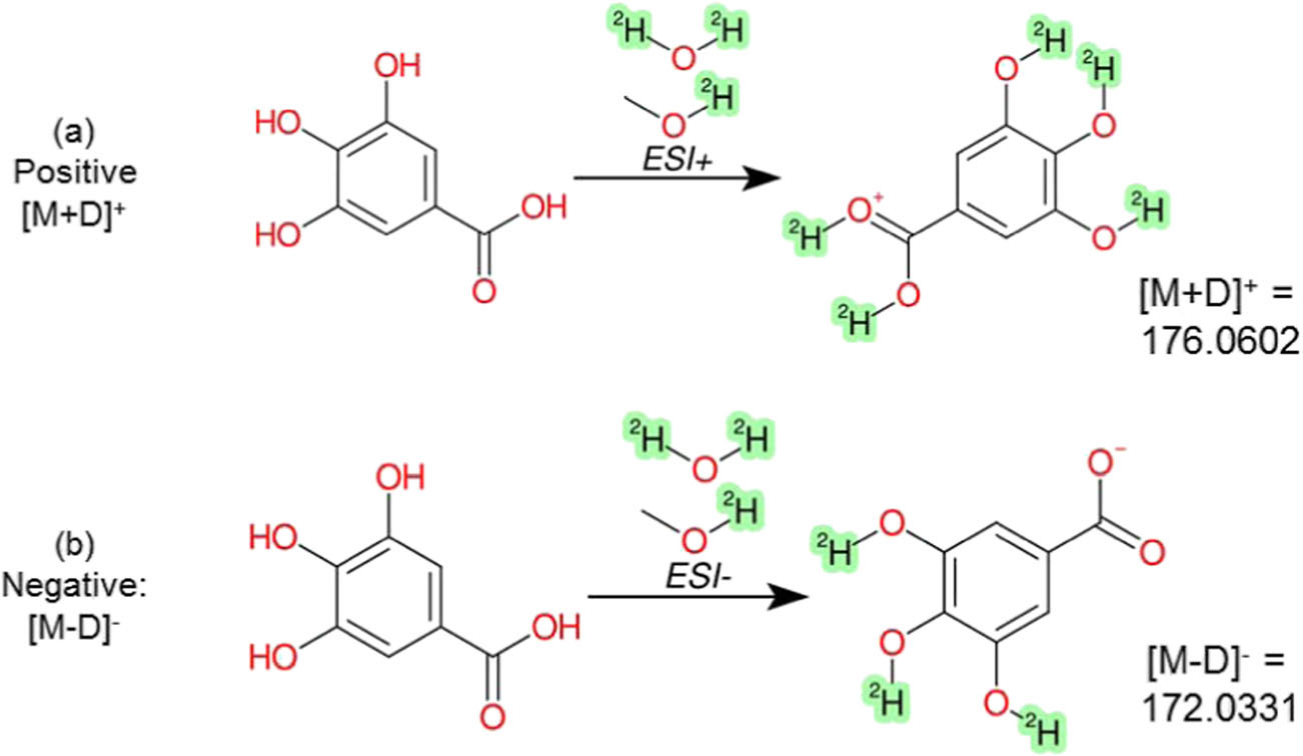
Example of expected HDX behavior of gallic acid (DTXSID0020650) in the experiment performed here in **a** positive ESI mode to produce [M+D]^+^ and **b** negative ESI mode to produce [M-D]^−^, along with the calculated ion masses that were subsequently observed in the experimental measurements. The quadruply deuterated species of gallic acid is available here (DTXSID60892625). Images created using CDK Depict [27] with SMARTS highlighting to indicate the deuterium. Note that while we refer to deuterium as “D” throughout the manuscript for simplicity, the depiction with ^2^H here is consistent with the standard representation of isotopes and enables the SMARTS-based highlighting shown

The base hypothesis to test was that “easily exchangeable” hydrogens would be exchanged in these experiments; thus, for all 762 substances, a prediction was made to exchange each heteroatom hydrogen with a deuterium (i.e., SH to SD, OH to OD, NH_2_ to ND_2_). The predicted deuterated formula was then used as a basis to search for deuterated spectra. In terms of the expected mass for each ionization mode, it was assumed that [M+D]^+^ ions would be formed in positive mode and [M-D]^−^ in negative mode (see “Introduction”). An example is given in Fig. 2 and further details are given in the “Implementation” section below. Note that while deuterium is commonly represented as “D,” a convention that we use in the text in this article for readability and consistency, the chemical representation used in the depictions is the isotopic form ^2^H, which allows for proper interpretation in the cheminformatics toolkits. The predicted deuterated SMILES for all substances are given in ESM Table S3d (note this is the prediction and not all species were observed). These predicted SMILES were used to perform the HDX data extraction (see next section). All observed (and manually verified) HDX features, given in ESM Table S3e-f, were registered in DSSTox, the database behind the CompTox Chemicals Dashboard [26], based on the predicted SMILES and mappings to the original standards. DSSTox was used to generate the remaining structural information presented in ESM Table S3f. The corresponding DSSTox substance identifiers (DTXSIDs) were used to create the HDXNOEX and HDXEXCH lists of undeuterated and deuterated species.

#### MS data processing

The raw data files from Thermo were converted to mzML using a front-end for MSConvert (from ProteoWizard [28]) written by U. Schmitt (SIS, ETHZ), using vendor centroiding, zero value removal, and zlib compression. The MS/MS of the standards were extracted using RMassBank [29]. The “normal” runs were processed in the typical RMassBank workflow, using the SMILES code for each chemical. As RMassBank could not (initially) handle deuterium when the data was extracted (due to issues with the Chemistry Development Kit that have subsequently been resolved [30]), the HDX data were extracted using the exact mass only, which meant that recalibration and noise removal was not performed on these data. Retention times (RTs) from the normal data were used initially, with a window of 0.4 min. Substances with RTs that were unknown were extracted using the RT at maximum EIC intensity for the precursor mass; for multiple peaks, these were determined manually. All RTs were checked manually and refined where necessary for those substances with missing results. For the normal runs, peak annotation and reanalyzed peaks options were both “true.” The recalibration was performed using loess fitting (see [29]) on assigned fragments and the MS1 data, using dppm. The MS1 and MS/MS were recalibrated together, with an initial window of 15 ppm. The multiplicity filter was set to 1 (as only one spectrum was recorded). All additional settings were the default ones (see file online). The extraction of the MSMS data was checked both visually and using a summary of the data (see Figures and Tables in the ESM). InChIKeys were used to check for duplicate chemical structures, while the spectral hash (SPLASH) [31] was used to detect identical extracted spectra for different substances. Data processing was all performed in the R programming language unless explicitly mentioned elsewhere.

### Implementation of HDX in MetFrag

MetFrag is a Java-based in silico fragmenter that uses the Chemistry Development Kit (CDK) [30, 32, 33] to read, write, and process chemical structures. The candidates are generally retrieved from compound databases using the neutral monoisotopic mass (calculated from the precursor) and a given relative mass deviation, the neutral molecular formula of the precursor or a set of database-dependent compound identifiers. Further details on MetFrag are given elsewhere [5, 34].

**Fig. 3:**
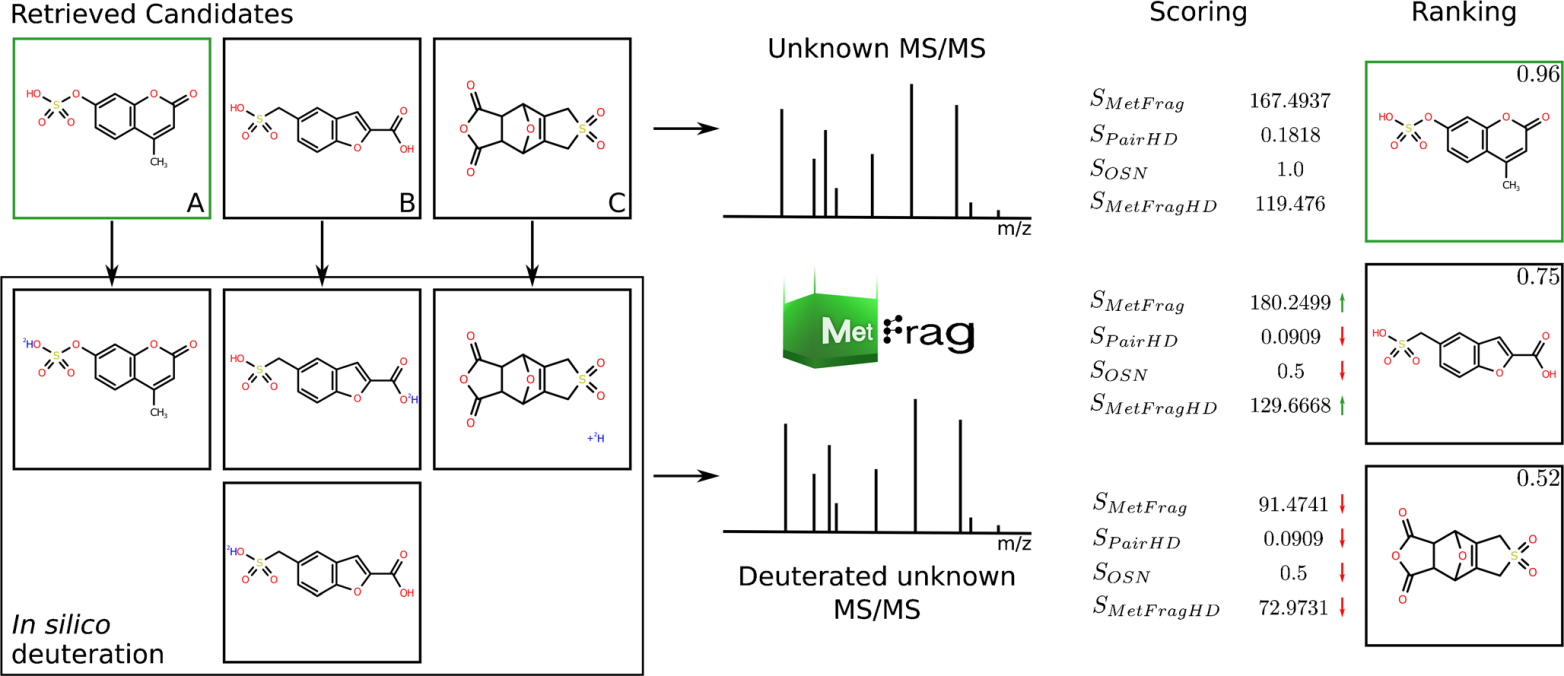
Workflow for MetFrag to analyze deuterated MS/MS spectra using the example of 4-methylumbelliferyl sulfate (**a**, green border) of the large standard set. The mass difference of the determined *neutral* precursor masses of the normal (256.0042 Da) and the deuterated (257.0104 Da) spectrum indicated X = 1, i.e., one exchanged hydrogen as shown for (**a**).Two additional selected candidates (**b**, **c**) illustrate different in silico deuteration cases where the retrieved candidate can result in two deuterated candidates (**b**) or one candidate of variable deuterium location as no easily exchangeable H is present (**c**). Processing normal and deuterated candidates with MetFrag-HDX results in four scoring terms for each candidate, which are combined in a consensus score using weight parameters retrieved during the cross-validation (~ 0.109, ~ 0.004, 0.497, ~ 0.39; see Methods; note, scores are normalized to range [0, 1]). This resulted in a top 1 ranking of the correct candidate 4-methylumbelliferyl sulfate. Green and red arrows mark scores that are higher or lower compared to those of the correct candidate. Candidate **b** is the top scoring candidate using S_MetFrag_ alone (without HDX information). This example illustrates both the workflow and the benefit of the additional scoring terms

The starting point for performing MetFrag on HDX data is the acquisition of two independent LC-MS/MS runs of one sample, where the first sample is acquired normally with undeuterated solvents (e.g., MeOH/H_2_O) and where at least one of the mobile phases is replaced with a deuterated equivalent during the second acquisition (e.g., MeOD/D_2_O, ACN/D_2_O). This yields two data sets and corresponding MS/MS spectra pairs (S_H_, S_D_) have to be collected where the precursor is in its normal form (“H”) and in its deuterated form (“D”), where S_H_ = {P_1_,...P_N_} contains N and S_D_ = {dP_1_,...dP_M_} M MS/MS peaks (middle part of Fig. 3). Each peak is defined by a *m/z* (mass to charge ratio) value m(P_N_) (for simplicity, we do not take into account intensities here). As reference standards were used in this manuscript, the expected deuterated species were predicted (based on the number of easily exchangeable Hs, as described above). These predicted masses were then used to extract the HDX MS/MS data, which was verified as described above. The undeuterated candidates were then deuterated in silico and matched to the experimental data, then combined using various scoring terms to yield the overall candidate rankings. Details on the generation and combination of these results are given below.

#### In silico deuteration of candidate structures

To use MetFrag’s in silico fragment generation for deuterated compounds, the algorithm was adapted to handle deuteriums as well as hydrogens. Furthermore, the MetFrag algorithm was extended to generate an in silico deuterated candidate list for a given MS/MS spectrum *S*_*D*_. First, MetFrag determines the number of experimentally exchanged hydrogens (*X*), which is calculated by the mass differences of the precursors of *S*_*H*_ and *S*_*D*_ as mentioned earlier. Given the candidate list *C* derived from a database search (e.g., PubChem [35], ChemSpider [36], or CompTox [26]), based on the precursor information (calculated monoisotopic mass, molecular formula) of the normal spectrum *S*_*H*_, MetFrag generates an in silico deuterated candidate list *dC*. For a candidate *C*_*i*_ ∈ *C*, the number of easily exchangeable hydrogens (*eH*(*C*_*i*_)) are determined by counting the number of hydrogens attached to oxygens, sulfurs, and nitrogens, namely hydroxyl/carboxyl, thiol, and amino groups. A graph-based approach is used to perform a simple search for the easily exchangeable Hs. During the method establishment, hydrogen/deuterium exchange was predicted assuming that all easily exchangeable hydrogens were 100% replaced with deuterium. This formed the “base case” for in silico deuteration and could be used to reject *C*_*i*_ as potential correct candidate in case (*eH ≠ X*). However, there are reasons why *eH*(*C*_*i*_) and *X* can differ, even when *C*_*i*_ is the correct candidate:

- Hydrogens attached to conjugated and/or aromatic carbons could be exchanged due to keto-enol tautomerism or by gas-phase reactions in the ESI source and thus the number of easily exchangeable hydrogens during measurement changes;
- easily exchangeable hydrogens might be stabilized by intramolecular hydrogen-bonding or sterically hindered; and
- the wrong isotopic peak was selected during data-dependent acquisition, leading to the wrong number of experimentally exchanged hydrogens (*X*).

Thus, different cases need to be handled for the in silico deuteration. Exactly one deuterated candidate is generated by exchanging all easily exchangeable hydrogens in case (*eH = X*)*.* Exactly one candidate is also generated in case (*eH < X*) by exchanging all easily exchangeable hydrogens of *C*_*i*_ and exchanging (*X - eH*(*C*_*i*_)) variable hydrogens (*vH*(*C*_*i*_)) of *C*_*i*_ assuming that also aliphatic and/or aromatic hydrogens are replaced without knowing the exact position (as the exact position of the Hs is not necessarily required explicitly during the fragmentation). Where (*eH*(*C*_*i*_) *> X*), MetFrag generates every combination of deuterated candidates where *X* out of *eH*(*C*_*i*_) easily exchangeable hydrogens are exchanged by deuterium, which results in (*X* choose *eH*(*C*_*i*_)) deuterated candidates for *C*_*i*_. Figure 3 shows example candidates for all three cases. This approach uses a modified version of the method used for in silico derivatization in [19]. The in silico deuteration method is available as a jar file and included as ESM. The predicted candidates are given in ESM Table S3d.

#### Scoring terms

To incorporate the information gained by additional deuterated experimental MS/MS spectra, different scores are calculated by MetFrag. Altogether, MetFrag calculates four scoring terms for a candidate *C*_*i*_ that are combined into a final (consensus) score. The regular *FragmenterScore* (*S*_MetFrag_(*C*_*i*_)), already introduced in [5], calculates the match of in silico–generated fragments *Frag*_*i,n*_ of a candidate *C*_*i*_ to the experimental MS/MS peaks *P*_*n*_ of *S*_*H*_, taking into account the relative intensity of a matched MS/MS peak, the *m/z* value, and the sum of the bond dissociation energies (BDEs) of the candidate bonds that were cleaved to generate the matching fragment.

**Fig. 4:**
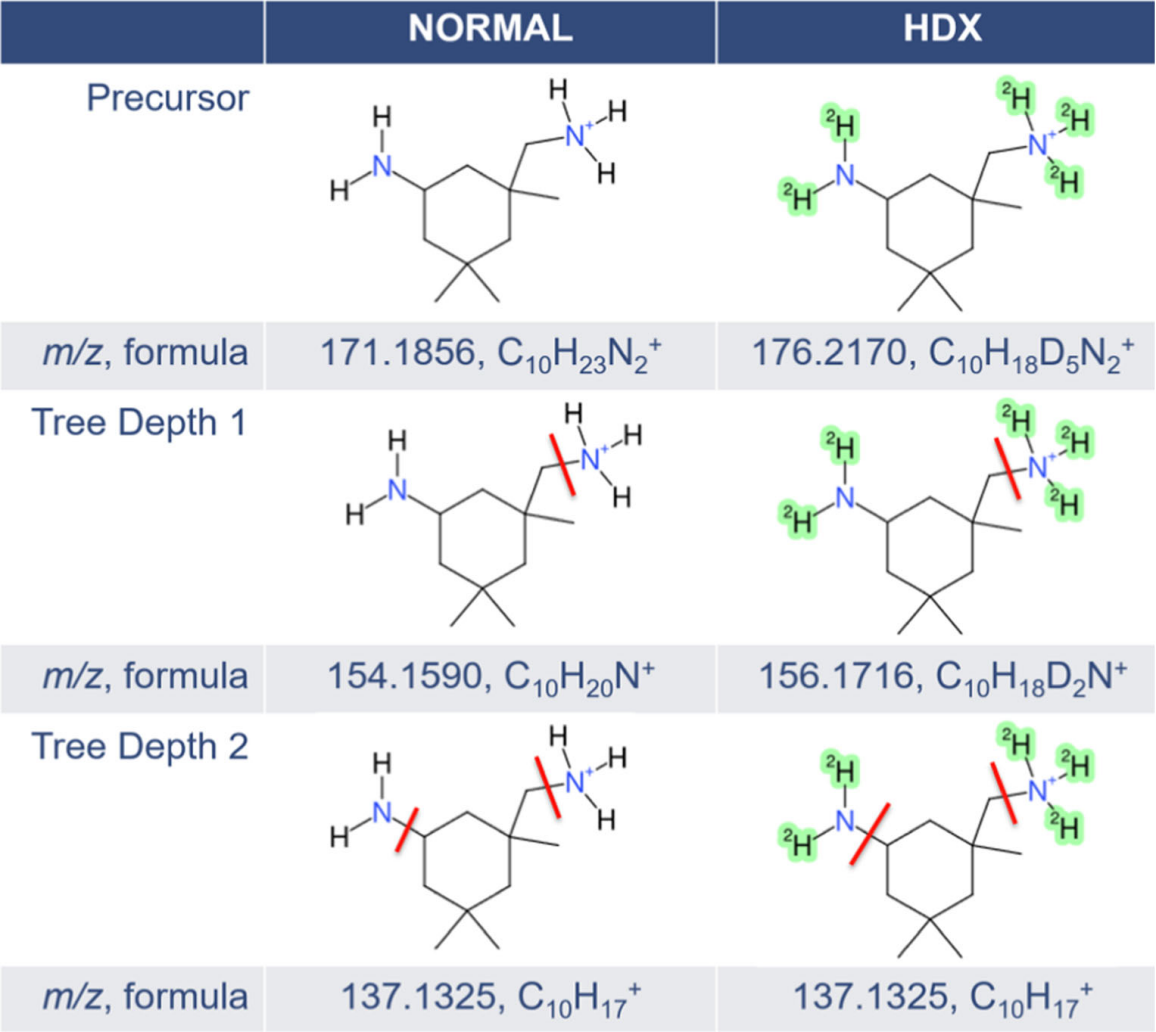
Modified in silico fragmentation workflow, demonstrated on isophorone diamine (DTXSID6027503). In silico–generated fragments from normal mode (left) are modified by exchanging and adding deuteriums at predicted positions (right, green shading) from the precursor molecule. The normal precursor is used to determine possible positions of hydrogen/deuterium exchange (here the amino groups). This information is used during the in silico fragmentation to perform mass calculation of deuterated fragments (left)

The *HDFragmenterScore* (*S*_MetFragHD_(*C*_*i*_)) uses the same calculation rule as the regular *FragmenterScore* with the same generated fragments but incorporates the information of exchanged hydrogens from the precursor candidate *C*_*i*_. This information is used to adapt calculated fragment masses to match against *m/z* peaks d*P*_*m*_ from the deuterated MS/MS spectrum *S*_*D*_ as illustrated in Fig. 4. The mass of a deuterated fragment dFrag_*i,n*_ is then calculated as

1 $$ m\left({\mathrm{dFrag}}_{i,n}\right)=m\left({\mathrm{Frag}}_{i,n}\right)+ eH\left({\mathrm{Frag}}_{i,n}\right)\cdotp \left(m(D)-m(H)\right); $$

where *m*(Frag_*i,n*_), *m*(*H*), and *m*(*D*) are the masses of the normal fragment, a hydrogen, and a deuterium, respectively.

Equation 1 simulates the exchange of a number *eH*(Frag_*i,n*_) of easily exchangeable hydrogens with deuterium for the related fragment. Where *vH*(*C*_*i*_) *≠* 0, MetFrag also tries to find a match based on a variable number of exchanged hydrogens by adapting fragment masses with

2 $$ m\left(\mathrm{d} Fra{g}_{i,n}\right)=m\left(\mathrm{d} Fra{g}_{i,n}\right)+k\cdotp \left(m(D)-m(H)\right); $$

where 1 ≤ *k* ≤ *vH*(*d*Frag_*i,n*_) to simulate an additional exchange of non-easily exchangeable hydrogens. As for the mass of the normal fragment Frag_*i,n*_, the adduct mass value *c* is added/subtracted also for *d*Frag_*i,n*_, which is usually the mass of a proton in the undeuterated case and thus the mass of *D*^*+*^ for the deuterated case.

The *HDFragmentPairScore* (*S*_PairHD_(*C*_*i*_)) counts matching fragment pairs (Frag_*i,n*_, *d*Frag_*i,n*_) between the normal and deuterated MS/MS spectrum. If a fragment Frag_*i,n*_ matches a peak in the normal MS/MS spectrum *S*_*H*_ and the corresponding deuterated fragment *d*Frag_*i,n*_ matches a peak in the deuterated MS/MS spectrum *S*_*D*_, it will be counted as a valid pair. For the matched MS/MS peaks *P*_*n*_ ∈ *S*_*H*_ and d*P*_*m*_ ∈ *S*_*D*_, the number of exchanged hydrogens *k* can be calculated by

3 $$ \mid m\left({P}_n\right)+k\cdot \left(m(D)-m(H)\right)-m\left(d{P}_m\right)\mid \le \in $$

where *є* is a predefined mass deviation and k ≤ X. A fragment pair is only counted if the number of deuteriums of *d*Frag_*i,n*_ are equal to *k*, so

4 $$ eH\left(\mathrm{d} Fra{g}_{i,n}\right)+ vH\left(\mathrm{d} Fra{g}_{i,n}\right)=k; $$

with 0 ≤ k, where a pair is also counted, if *k = 0* and *eH*(*d*Frag_*i,n*_) *+ vH*(*d*Frag_*i,n*_) *= 0* meaning *dFrag*_*ij*_ carries no deuterium.

The *HDExchangedHydrogensScore* (*S*_OSN_(*C*_*i*_)) shown in Eq. 5 boosts candidates whose predicted number of easily exchangeable hydrogens *eH*(*C*_*i*_) matches the number of experimentally exchanged hydrogens *X* and discriminates those the more the higher the two values deviate from each other assuming that all and only easily exchangeable hydrogens are exchanged in most of the cases.

5 $$ {S}_{Ci(OSN)}=1/\left(|X- eH\left({C}_i\right)|+1\right)\ \Big) $$

The four scoring terms are calculated for all candidates *C*_*i*_ in the candidate list *C* and are normalized by the maximum value within *C*. The final score, which is used to rank the candidates *C*_*i*_, is calculated by the weighted sum (represented by the respective weighting terms *ω*), as shown in Eq. 6.

6 $$ {S}_{Ci}={\omega}_{MetFrag}\cdotp {S}_{MetFrag}\left({C}_i\right)+{\omega}_{MetFrag HD}\cdotp {S}_{MetFrag HD}\left({C}_i\right)+{\omega}_{PairHD}\cdotp {S}_{PairHD}\left({C}_i\right)+{\omega}_{OSN}\cdotp {S}_{OSN}\left({C}_i\right) $$

In case more than one deuterated candidate exists for a given candidate *C*_*i*_, the maxima of *S*_MetFragHD_(*C*_*i*_) and *S*_PairHD_(*C*_*i*_) over the generated deuterated candidates are used for Eq. 6.

#### Evaluation and optimization

To test the workflow, the adapted MetFrag algorithm was used to process all spectra pairs from sets 2 and 3. Candidates were retrieved by querying the ChemSpider database (June, 2017) with the formula of the correct precursor molecule. Candidates consisting of non-covalently bound substructures (e.g., salts) and containing non-standard isotopes (like ^13^C) were filtered out and not considered for the final scoring. For the processing of the normal and deuterated MS/MS peak lists, a relative and absolute mass deviation of 5 ppm and 0.001 Da was used for set 3 and 10 ppm and 0.01 Da for set 2 to match in silico*–*generated fragments to experimental MS/MS peaks. MetFrag calculated the four scoring terms *S*_MetFrag_(*C*_*i*_), *S*_MetFragHD_(*C*_*i*_), *S*_PairHD_(*C*_*i*_), and *S*_OSN_(*C*_*i*_) for each of the candidates. The weights *ω*_MetFrag_, *ω*_MetFragHD_, *ω*_PairHD_, and *ω*_OSN_ were optimized by a randomized grid search for which 1000 weight combinations were drawn uniformly from the simplex. The optimal weight combination was determined by maximizing the number of correctly top 1 ranked candidates among the MS/MS spectra pairs in the training set. In case several candidates shared the same final score as the correct one, the average rank was reported. Prior to the ranking, duplicate entries within the candidate list were filtered based on the first part of the candidates’ InChIKey. The optimization was performed by a tenfold cross-validation for the large standard set (set 3) with a randomized fold assignment of the spectra pairs. Due to a lower number of spectrum pairs, a leave-one-outcross-validation was used for set 2. To determine the influence of the scoring terms on the ranking results for set 3, the same cross-validation (same fold assignment) was repeated by considering different sets of scoring terms used to calculate the final score *S*_*Ci*_. The term combinations considered were {*S*_MetFrag_(*C*_*i*_), *S*_MetFragHD_(*C*_*i*_), *S*_PairHD_(*C*_*i*_)}, {*S*_MetFrag_(*C*_*i*_), *S*_MetFragHD_(*C*_*i*_), *S*_OSN_(*C*_*i*_)}, and {*S*_MetFrag_(*C*_*i*_), *S*_MetFragHD_(*C*_*i*_)}.

## Results

### Set 1: Fragmentation of deuterated standards

To extend MetFrag to deal with deuterium, MS/MS spectra of three deuterated (internal) standards (where the location of deuterium is known and not expected to undergo any form of exchange during the experiment) were extracted using RMassBank and compared with QExactive spectra of the corresponding undeuterated substances available in MassBank. The three standards (DEET and DEET-d7, metolachlor and metolachlor-d6, carbamazepine and carbamazepine-d10) are shown in ESM Table S1, along with database identifiers and the corresponding best-matching MassBank spectrum. Table S4 (see ESM) shows the two main example fragment pairs from DEET and DEET-d7, with formulas as annotated by MetFrag and proposed fragment structures. The corresponding MS/MS spectra are given in ESM Fig. S1.

The spectrum of metolachlor-d6(see ESM Fig. S2) revealed more interesting fragmentation information than DEET for the MetFrag results, as the deuteration was for only 6 of the total 22 hydrogens. As expected, the undeuterated fragment C_4_H_9_O^+^ at *m/z* 73.0648, lost from the nitrogen, was observed as C_4_H_3_D_6_O^+^ at *m/z* 79.1022 for metolachlor-d6(see ESM Table S1). Corresponding *m/z* fragments prior to the loss of this group were also seen, e.g., C_12_H_18_N^+^ (*m/z* 176.1434) in the undeuterated molecule and C_12_H_12_D_6_N^+^ (*m/z* 182.1815) in the deuterated molecule. However, many fragments associated with the aromatic group (originally undeuterated) were also observed incorporating one or more deuteriums. This indicates that the replacement of Hs with Ds can also occur at the aromatic ring in the collision cell, either due to rearrangement reactions involving a movement of Ds in activated gas-phase ions (scrambling) or an exchange with other species present in the cell [37, 38]. Examples observed at high intensities in the MS/MS spectra included C_7_H_7_^+^ (*m/z* 91.0542) to C_7_H_6_D^+^ (*m/z* 92.0603); C_6_H_7_N^+^ (*m/z* 93.0573) to C_6_H_6_DN^+^ (*m/z* 94.0632) and C_6_H_5_D_2_N^+^ (*m/z* 95.0698); C_7_H_10_N^+^ (*m/z* 108.0807) to C_7_H_9_DN^+^ (*m/z* 109.0872) and C_7_H_8_D_2_N^+^ (*m/z* 110.0933). The most important conclusion from this exercise for MetFrag, apart from the successful method development, that this mobile deuterium in the collision cell should be considered dynamically, similar to hydrogen [5], i.e., fragments can be explained with up to one or two additional hydrogens or deuteriums.

### Set 2: QToF HDX experiments

The spectra from this test set, although a minor contribution in comparison to the larger standard set described below, were invaluable in establishing and testing the scoring strategy implemented in MetFrag before the complete large standard set was available. However, the results do illustrate the impact of lower mass accuracy in HDX as obtained by the used QToF instrument. The results retrieved for selected compounds are given in ESM Table S2 along with the structures and the weights of the scoring function and the resulting ranks. The candidates were retrieved with a ChemSpider query as described above. The top row per compound contains the results considering only MetFrag without the deuterated scoring terms, while the lower two rows show results with different weightings (given in ESM Table S2) of all terms. The table shows clearly for each example that the candidate ranking and thus the results are improved when considering the information from the deuterated experiments. Drastic improvements are obtained for the examples N-(3-indolylacetyl)-L-valine and phlorizin where the rankings improved from 97 to 25 and from 14 to 3.5, respectively. While the original results for this test set actually eliminated candidates that exchanged fewer H atoms, subsequent testing revealed that this could potentially result in the elimination of correct candidates. As a result, the methods were adjusted to the final strategy presented in this publication, where all candidates are scored and the scores are used to provide relative rankings, rather than performing a hard elimination of any candidates not exactly matching the theory. All further validation was performed on the large standard set, described below, as this was a much more comprehensive dataset and the greater substance numbers were required for a more comprehensive evaluation of the method.

### Set 3: Evaluation on large standard set

#### Experimental results on large standard set

As described in the methods, several mixtures were measured to obtain the experimental data for the HDX method development and validation. Several re-measurements were undertaken to confirm observations and obtain the highest quality MS/MS spectra possible. In total, pairs of spectra (i.e., valid MS/MS spectra in both normal and HDX measurements) were found for 592 of the 762 unique substances measured. As described in the methods, these were quality controlled with automated curation, control checks, and automated plotting of extracted spectra and spectral pairs. All spectra were verified manually by at least two of the authorship team, including cross-checks in the vendor software. The results generally matched very well with the theory explained above, and were overall better than anticipated given the large structural diversity and myriad of functional groups and properties in this large standard set. An overview of all observed retention times plus respective columns and measurement is given in ESM Table S3e. The chemical information associated with all of these observed species, including number of deuteriums exchanged and deuterated structures (where applicable), is given in ESM Table S3f. These observed structures are available for readers to download (https://comptox.epa.gov/dashboard/chemical_lists/hdxexch). The full substance listing is also available at https://comptox.epa.gov/dashboard/chemical_lists/hdxnoex (reference standards only, not including the deuterated species).

**Fig. 5:**
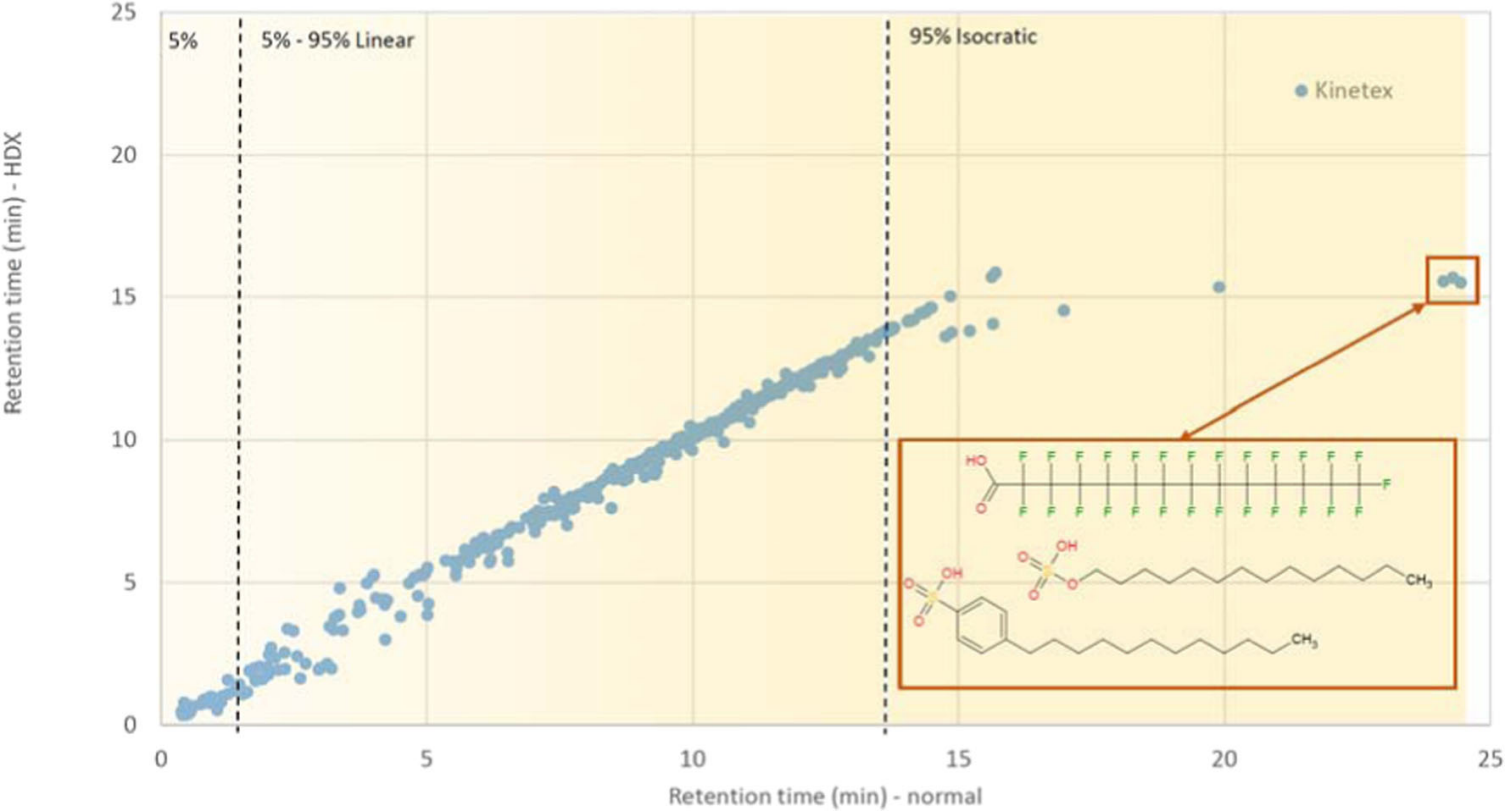
Retention time (in minutes) of all (unique) substances detected in normal (x axis) and HDX (y axis) conditions for the substances measured on the Kinetex column (both ESI positive and negative modes). The gradient and percentage of methanol (normal) are marked with yellow highlighting and dashed lines. Examples for the extreme retention time shifts observed are given in the box and in ESM Fig. S4; for explanations, see text

Example chromatograms (one normal, one HDX, ESI positive mode) for the pesticide mix are given in the ESM (Fig. S3). This shows that overall, the chromatograms look similar in many places, although peaks are clearly shifted slightly (sometimes lower, sometimes higher retention times—for instance, 5.51 to 5.80 min and 13.46 to 13.36 min in normal and HDX conditions, respectively). In the isocratic region (after approx. 15 min), peaks appear at much higher intensity in the HDX chromatogram than in the normal chromatogram for the Kinetex column—a phenomenon that was reproducible in both the standard mixes and environmental samples (discussed further below). The normal vs HDX retention times over all mixes for the final compiled dataset are plotted in Fig. 5 for the Kinetex column. The retention times are generally on the 1:1 line (with some small deviations at very early retention times) until approximately 13 min, where the elution regime changes from gradient to isocratic with 100% MeOH/MeOD, respectively. Several compounds are still on the 1:1 lineup to 16 min, while others deviate markedly from this trend, eluting up to 25 min in normal mode but by 16 min in HDX. The latter structures were all surfactants with a polar head group and a long, apolar tail. Two of the most extreme examples are dodecylbenzenesulfonic acid (DTXSID8050443) and perfluorotetradecanoic acid (DTXSID3059921), as shown in Fig. 5. Despite these few extreme examples, the average retention time shift over all standards was 0.04 min. A figure showing the retention time vs change in retention time between the columns is included in the ESM (Fig. S4), including additional example structures for standout data points. While the change in physicochemical properties from the normal to the deuterated eluents hardly affects the compound retention during the relatively steep gradient elution, these differences have a much larger effect on surfactants during the isocratic elution. For the Synergi column, the average retention time shift was 0.35 min, but note this was only for 45 substances measured with a long chromatographic gradient to enable better separation.

**Fig. 6:**
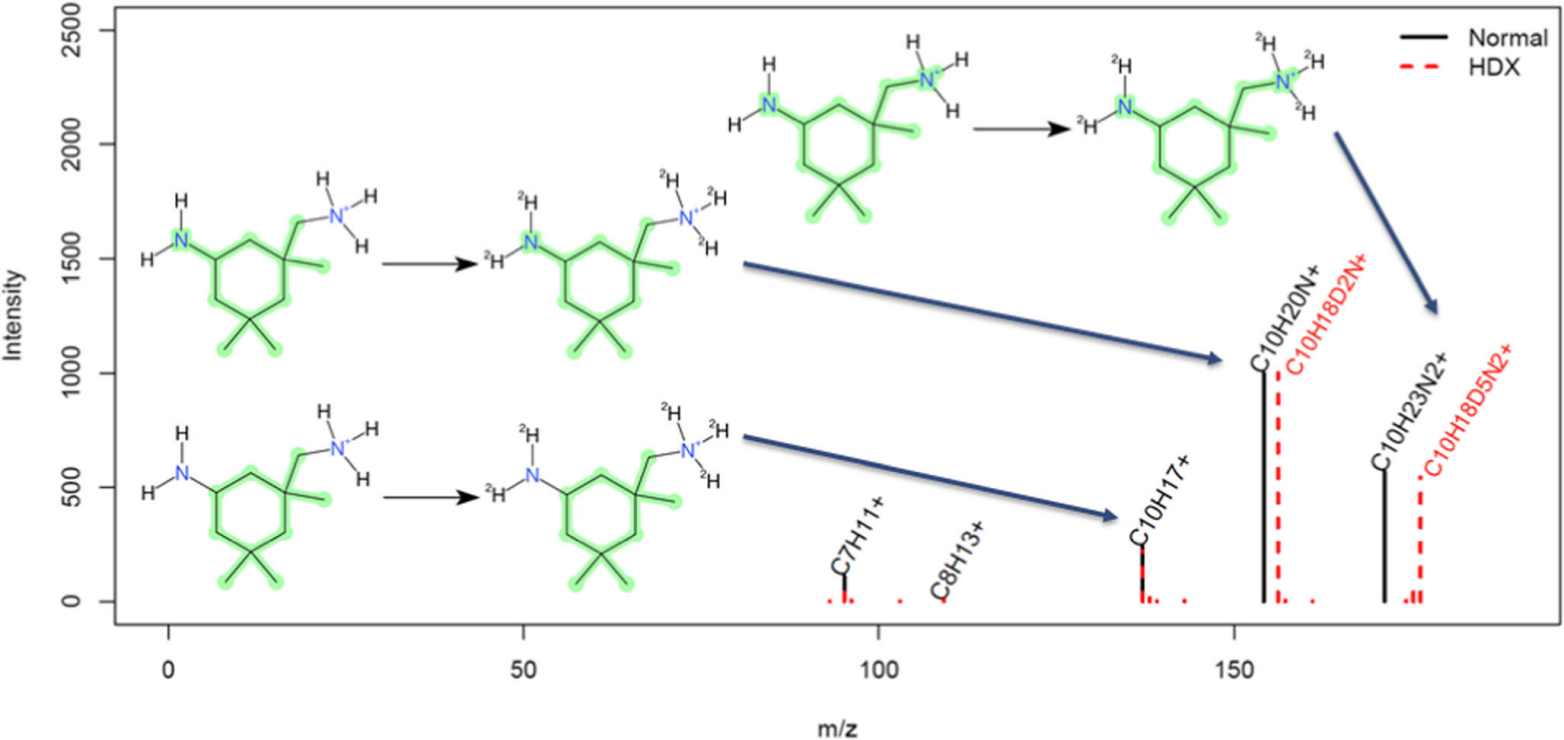
Observed normal (black) and HDX (red dashed) MS/MS fragments for isophorone diamine (DTXSID6027503) showing the [M+D]^+^ ion (shifted by 5 mass units, as expected when 4D are exchanged plus an additional D is gained in ionization), then a NH_3_/ND_3_ loss to yield a fragment pair with a 2 mass unit shift, then a subsequent NH_2_/ND_2_ loss to yield the identical C_10_H_17_^+^ fragment with no more deuterium present. Images created using CDK Depict; the highlighting indicates the remaining “backbone” of the structure, as represented in MetFrag

The majority of MS/MS spectra, 505 pairs, were found in positive ion mode, while 155 pairs of spectra were found in negative ion mode (68 substances had pairs in both modes). A summary of the MS/MS information is given in ESM Table S3g. While fewer substances ionize in negative mode, there was also a significant loss of intensity in the negative mode HDX spectra (reproducible across several measurements) that contributed to the significantly lower proportion of negative mode pairs. While intensity losses were also observed in positive mode, the generally higher intensity values in positive ESI resulted in many more spectral pairs in positive mode. The average maximum intensities across the MS/MS acquired from the major three chromatographic runs (first measurements and bulk re-measurements on the Kinetex column plus the Synergi runs) were 2.21 × 10^8^ for positive normal, 1.03 × 10^8^ for positive HDX (both over 499 observations), 1.75 × 10^7^ for negative normal, and 9.57 × 10^6^ for negative HDX (over 153 observations). The highest maximum intensities observed in the MS/MS (in the same order) were 4.7 × 10^9^, 2.1 × 10^9^, 2.4 × 10^8^, and 1.3 × 10^8^, while the lowest maximum intensity was 1.7 × 10^5^, 5.6 × 10^4^, 3.8 × 10^4^, and 2 × 10^4^. Based on experience, a maximum intensity above 1 × 10^5^ in the MS/MS is required (for this instrument) for a sufficiently informative spectrum; thus, part of the manual checks performed was to judge whether the extracted MS/MS were of sufficient intensity, and thus quality, for the purposes of this study. A further overall factor to consider was the number of fragments observed. The average number of fragments (same order as previously) was 30, 50, 11, and 28 fragments (see ESM Table S3g for a full listing). Note that while more fragments were observed for HDX (50 vs 30, 28 vs 11), this is both due to the potential for more fragments on account of the exchange behavior but also because a less rigorous cleanup was performed (see “Methods” section and Fig. 6 below). Furthermore, the presence of more fragments reduces the intensity of single fragments and this could partially explain the intensity losses observed in the HDX spectra. The maximum number of fragments observed was 267, 383, 104, and 112, respectively, with minimum 1 for all categories except negative HDX (5). Visual checks were performed to eliminate the presence of spectra that may just be noise or where the pairs appeared to completely mismatch, or where only peaks resulting from the precursor (or higher) were present, as these are not accounted for during MetFrag processing. Following all manual checks, 499 spectral pairs remained for positive mode and 148 for negative mode (see ESM Table S3g). This dataset formed the basis for the MetFrag Score validation (see next section).

In the end, matching pairs were observed as one or both of [M+H]^+^/[M+D]^+^ and [M-H]^−^/[M-H/D]^−^ for 592 of the original 762 substances (78%) and 579 (76%) of these were used further for method development following manual checks. For 170 substances, no valid pairs were observed for a number of reasons, which are clarified in the following examples. It is possible that some “pairs” have been falsely eliminated in the quest for optimal data quality. For instance, in positive mode, retention times were determined for 656 of 850 (non-unique) [M+H]^+^ species over the two major runs of all mixes, whereas only 631 RTs could be determined for the equivalent [M+D]^+^species—in the vast majority of cases due to lack of intensity, poor peak shape or evidence of interfering co-elution. Overall, very little evidence of partial or incomplete exchange was observed. For negative mode, retention times could be determined for 206 [M-H]^−^ species and 195 [M-H/D]^−^ species according to the theory described in the methods; no substances exhibiting partial exchange were noted, but as stated above, the intensity losses in negative mode made it difficult to find valid pairs in some cases. A few substances were not extracted due to incorrect structural information in the original compound lists used to perform the data extraction (i.e., SMILES and name mismatch, which only became obvious during quality control)—while the tables presented in ESM Table S3 have been extensively curated and present the correct structural information to the best of our knowledge, the spectra were not re-extracted from the raw data for the cases where these errors were discovered too late and resulted in the wrong masses and wrong predicted structures, etc. A further case resulting in the most “non detects” for positive mode was the formation of adducts other than [M+H]^+^, resulting in the loss of 13 substances expected as [M]^+^ and another (Abamectin) observed almost exclusively as [M+Na]^+^ and [M+NH_4_]^+^. Although MetFrag can handle different adduct settings, for the purpose of simplicity for the method establishment here (and due to the low number of adducts observed resulting in very small datasets), it was decided to evaluate the [M+H]^+^/[M+D]^+^ and [M-H]^−^/[M-D]^−^ cases only in the material presented here. Alternative adducts were not investigated in negative mode due to the intensity issues, which made it difficult to draw any form of conclusion. As measurements were performed on several mixes rather than individual compounds, it is also worth noting that these mixtures were chosen partially for analytical convenience and many substances present in some mixes would require a more specialized chromatography for optimal measurement (e.g., many steroids and amines) and it was not expected that all substances would be observed in these experiments. This compromise was necessary to obtain the data presented here, as flooding a complete chromatographic system with deuterated solvents leads to an approximately 50 times cost increase per run above regular solvents (see discussion below).

The results achieved exceeded expectations in many ways and many high-quality normal and HDX spectra were obtained. As an example, the observed spectra (normal and HDX mode) for isophorone diamine, DTXSID6027503, are shown in Fig. 6 (a small compound has been chosen for clarity). The fragmentation is explained in the figure and caption.

#### MetFragHDX score validation

As described in the “Methods” section, four scoring terms were considered to account for the additional information arising from HDX experiments in MetFrag (see Eq. 6). The final selection of MS/MS pairs (as described above) was used in the evaluation of the scoring terms (note that a total of 498 spectra were used in positive mode as one compound was measured on both columns). The results are given in Table 1. The improvement in rank was much clearer for set 3, where the Top 1 ranks increased from 49 (10%) using the original MetFrag scoring alone to 78 (16%) by incorporating HDX information for the positive mode spectra. The results in Table 1 were also visualized to gain an overall view of the candidate ranking improvement. While in some cases using only three of the four terms yielded similar ranking results, in the end, all four terms were retained as each contributes valuable information for the interpretation of the results. Furthermore, the MetFrag output is designed in such a way that users can access all individual scoring terms in the results export and are thus able to re-score the results (or exclude specific terms) at any stage using their own weighting scheme.

**Table 1:** Absolute number (%) of top 1, 3, 5, and 10 ranks for MetFragHDX Score combinations for set 2 (57 and 63 MS/MS spectra) and set 3 (498 and 147 spectra) in positive and negative modes respectively. Results for all score terms and MetFrag only are shown for set 2; various combinations for set 3. Although some of the individual scores do not have good ranking performance, the combination of all 4 terms results in a clear improvement. The combination of all four terms outperformed the tested combinations of 2–3 terms

Set 2 (QTOF)	Positive (*n* = 57)				Negative (*n* = 63)			
	Top 1	Top 3	Top 5	Top 10	Top 1	Top 3	Top 5	Top 10
MetFrag,PairHD,OSN,MetFragHD	4 (7%)	9 (16%)	15 (26%)	24 (42%)	2 (3%)	13 (21%)	19 (30%)	31 (49%)
MetFrag	4 (7%)	8 (14%)	11 (19%)	13 (23%)	1 (2%)	4 (6%)	5 (8%)	14 (22%)
Set 3 (Orbitrap)	Positive (*n* = 498)				Negative (*n* = 147)			
	Top 1	Top 3	Top 5	Top 10	Top 1	Top 3	Top 5	Top 10
MetFrag,PairHD,OSN,MetFragHD	78 (16%)	189 (38%)	251 (50%)	320 (64%)	20 (14%)	64 (44%)	90 (61%)	106 (72%)
MetFrag,PairHD,OSN	74 (15%)	192 (39%)	254 (51%)	321 (64%)	20 (14%)	61 (41%)	86 (59%)	106 (72%)
MetFrag,MetFragHD,PairHD	56 (11%)	145 (29%)	197 (40%)	255 (51%)	15 (10%)	48 (33%)	74 (50%)	86 (59%)
MetFrag,MetFragHD,OSN	76 (15%)	191 (38%)	255 (51%)	322 (65%)	21 (14%)	67 (46%)	89 (61%)	107 (73%)
MetFrag,MetFragHD	59 (12%)	152 (31%)	202 (41%)	258 (52%)	18 (12%)	49 (33%)	68 (46%)	82 (56%)
MetFrag,PairHD	51 (10%)	146 (29%)	200 (40%)	250 (50%)	16 (11%)	49 (33%)	69 (47%)	84 (57%)
MetFrag,OSN	74 (15%)	193 (39%)	253 (51%)	320 (64%)	21 (14%)	62 (42%)	86 (59%)	107 (73%)
PairHD,OSN	30 (6%)	109 (22%)	154 (31%)	224 (45%)	12 (8%)	46 (31%)	68 (46%)	90 (61%)
MetFragHD,PairHD	56 (11%)	133 (27%)	189 (38%)	238 (48%)	13 (9%)	42 (29%)	61 (41%)	78 (53%)
MetFrag	49 (10%)	130 (26%)	177 (36%)	238 (48%)	18 (12%)	47 (32%)	61 (41%)	80 (54%)
PairHD	26 (5%)	82 (16%)	121 (24%)	165 (33%)	8 (5%)	33 (22%)	54 (37%)	68 (46%)
OSN	12 (2%)	52 (10%)	87 (17%)	137 (28%)	8 (5%)	28 (19%)	50 (34%)	71 (48%)
MetFragHD	55 (11%)	130 (26%)	180 (36%)	235 (47%)	13 (9%)	40 (27%)	60 (41%)	72 (49%)

### Observations on environmental sample

**Fig. 7:**
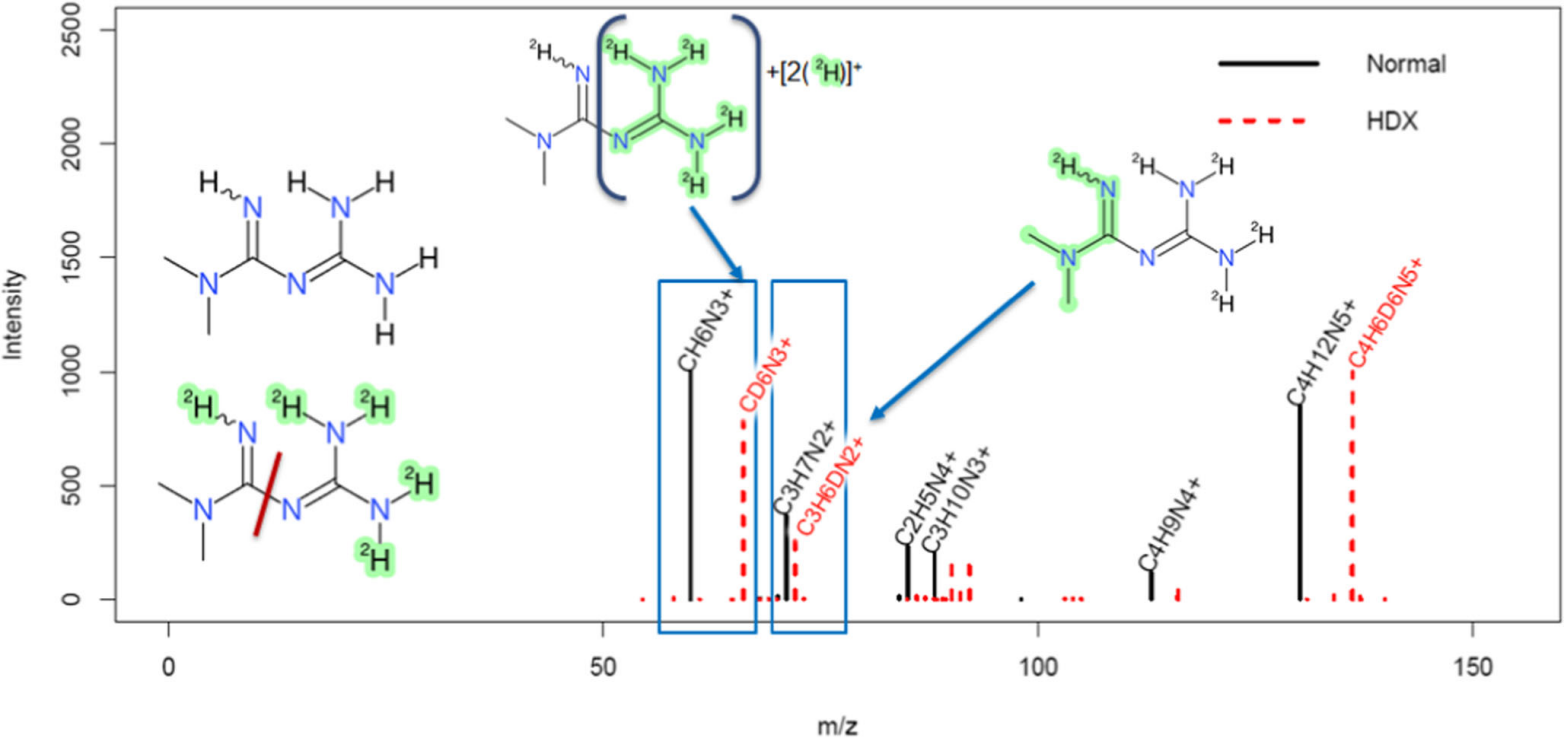
Metformin (DTXSID2023270) in the Novi Sad sample; black in normal conditions and red dashed as observed under HDX conditions. The shift of the major fragments clearly shows the origins of the fragments (see red line indicating the major “split” in the inset). Green highlighting in the fragments indicates the remaining backbone as represented in MetFrag

The same chromatographic methods (normal and HDX) were applied to an environmental sample to investigate how transferable these methods would be to “real world” samples. A well-characterized sample that was the focus of the joint EU project SOLUTIONS (https://www.solutions-project.eu/) was chosen (see “Methods”). Screenshots of the full scan chromatograms are given in the ESM (ESM Figs. S5 and S6, in positive and negative modes, respectively). The targeted analytical results performed on this sample [22] were used to confirm the results observed for the mixes (see ESM Table S5a). As an example, the MS/MS spectra for metformin are shown in Fig. 7 below, with the expected reaction and corresponding chromatographic peaks in ESM Fig. S7. For comparison, the corresponding normal and HDX spectra for metformin from the standard mixes (as opposed to the sample) are given in ESM Fig. S8; the spectral similarity between the HDX spectrum from the sample and the mix (without performing any form of additional spectral processing or cleanup) was 0.87, mainly due to the presence of additional peaks in the sample spectra.

In total, 107 target compounds that were reported were deemed to be detectable with the non-target Orbitrap method used here (many at low concentrations, see ESM Table S5). Of these 107, 90 pairs of normal and HDX peaks were found (68 in positive mode, 22 in negative mode), excluding messy or unclear peaks. MS/MS pairs existed for 28 of these (21 positive, 7 negative). For the remaining pairs, either no MS/MS was observed in normal conditions (6), under HDX conditions (27), or both (46). This is partially influenced by the data-dependent acquisition used (i.e., no inclusion list was used to try to record MS/MS spectra for these compounds, which would be a realistic scenario for performing non-target analysis on a sample with unknown compounds). These results are summarized in ESM Table S5a. The average intensities (for peaks where pairs were observed) were 3.5 × 10^7^, 2.4 × 10^7^, 3.3 × 10^6^, and 1.3 × 10^6^ for positive normal, positive HDX, negative normal, and negative HDX, respectively. The average retention time shift over both modes was 0.20 min.

**Fig. 8:**
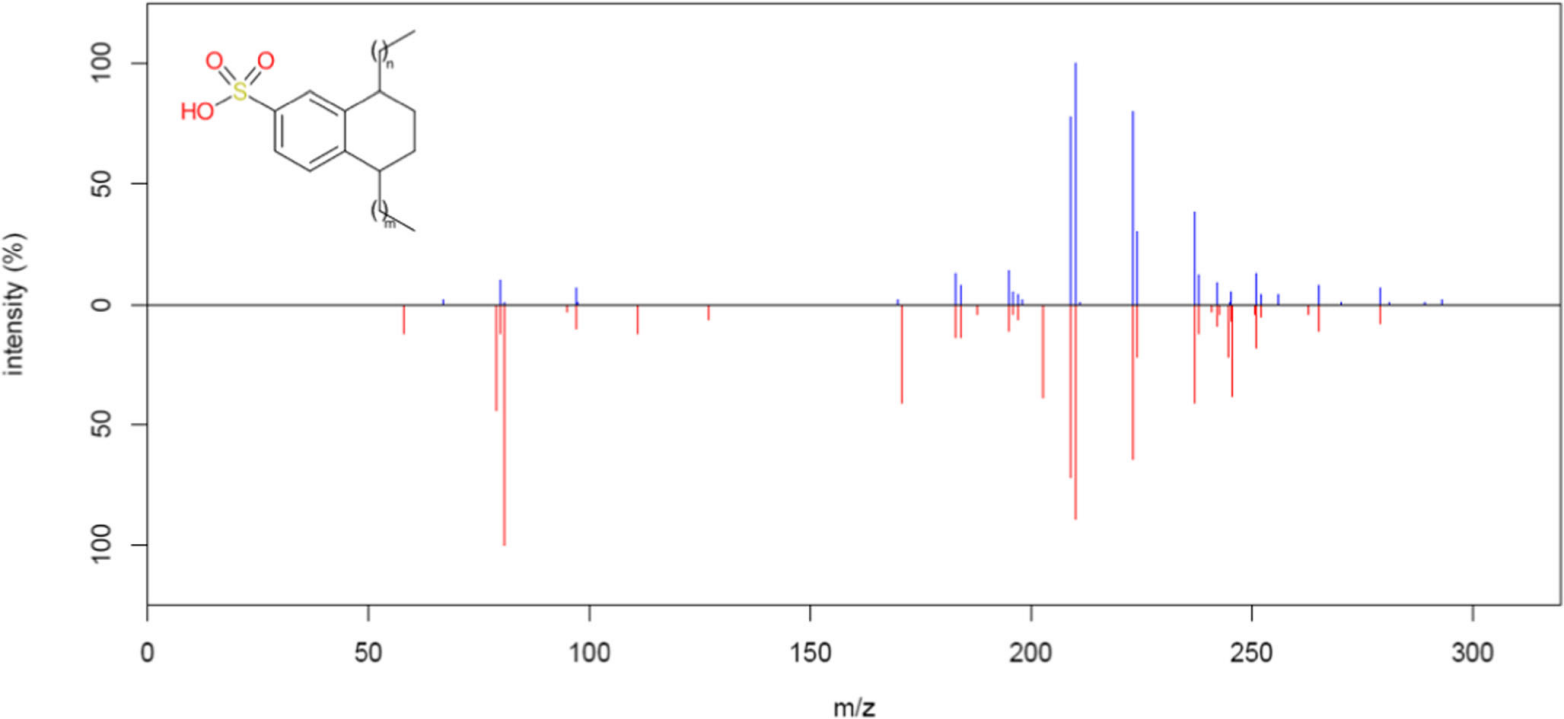
Head to tail plot of MS/MS fragments from C11-DATS (where m + *n* = 5) in the Novi Sad sample. Blue: normal; red: HDX fragmentation. As only 1 D can be exchanged, which is lost during ionization, no D is observed in the structure of the ion. Shifts in the peaks in the lower masses are still observed due to the presence of D in the collision cell interacting with the aromatic structure, likely arising from other (deuterated) precursor ions included within the isolation window. Note that the high-intensity precursor peaks (m/z 309.1530) have been excluded from both spectra to allow for better visualization of the fragmentation patterns. A lower intensity (~ 10%) precursor mass of m/z 308.6758 was observed in the full scan data for the HDX measurements, which would have been included in the isolation window for the HDX MS/MS data and could have been the source of deuterium. This mass was only visible at 2% in the MS/MS spectrum

As for the standard mixes, a significant loss in intensity was again observed for the negative mode HDX measurements (see ESM Fig. S6), except for substances occurring after the isocratic gradient at 13 min, which once again sharpened dramatically and substances eluted much earlier in HDX conditions. While the positive mode data appears visually similar (ESM Fig. S5), this is not the case for negative mode (ESM Fig. S6), where most of the visible peaks between 0.4 and 14 min in the normal chromatogram are no longer (or only very slightly) visible in the HDX chromatogram, while the unresolved lump towards the end, due to dialkyl tetralin sulfonate (DATS, DTXSID70891725) surfactants, among others, has sharpened to a family of peaks between 14.5 and 16 min. The chromatography associated with individual masses in this homologous series is demonstrated in ESM Fig. S9. The corresponding fragmentation spectra in normal and HDX mode for C11-DATS (C_17_H_26_O_3_S, precursor *m/z* 309.1530, identification level 3 [39]) is given as a head to tail plot in Fig. 8.

This retention time shift was also observed for the target compound perfluorooctanoic acid (DTXSID8031865), which was observed at RT = 15.5 min in normal mode and 13.7 min in HDX conditions. To investigate whether this is a phenomenon driven by the properties of these type of substances (a long apolar part followed by a polar head group), the sulfophenyl alkyl carboxylate (SPACs, DTXSID90891722) surfactants were also investigated, as these have polar functional groups on both ends of the molecule, due to the presence of the carboxyl group at the end of the alkyl chain. While these surfactants also suffered from the intensity loss in negative mode, they elute much earlier and did not appear to display large retention time shifts under HDX conditions (see ESM Fig. S10), although no MS/MS was obtained. Subsequently, surfactant series detected in wastewater [23], available here: https://comptox.epa.gov/dashboard/chemical_lists/eawagsurf, were screened by formula using RChemMass (https://github.com/schymane/RChemMass). Significant shifts were observed for tentatively identified (level 3) groups of AS surfactants (RT 22–25 min to 14–15 min), DATS (RTs 21–24 min to 12–15 min), LAS (> 24 min to 14–16 min). Less conclusive shifts, but clear sharpening of the elution profile in HDX mode, was observed for the AES and SAS classes, see ESM Table S5b.

## Discussion

This article describes the integration of hydrogen-deuterium exchange (HDX) experiments into MetFrag to assist in the identification of unknown compounds in non-target high-resolution mass spectrometry experiments. The initial algorithms were implemented and tested on a small subset of stably labeled deuterated substances to ensure correct handling of deuterium. The full method was then applied to small test sets of hydrogen-deuterium exchange experiments before being evaluated extensively on a large set of environmental standards and finally applied to an environmental sample. Thus, the methods presented here have been validated on two separate LC-MS systems, one Orbitrap-based, and another QTOF-based. The experimental results were, in many ways, better than anticipated. For the standard mixes, very little deviation from the expected exchange behavior was observed and, despite intensity losses in negative mode observed for the Orbitrap data, generally very comparable MS/MS were obtained. However, despite this, the ranking improvements were not as great as hoped on the large set of ChemSpider candidates, with an increase from 10 to 16% of the candidates ranked correctly in first place. This contrasts with the influence of metadata on candidate ranking in MetFrag observed in the CASMI2016 results, which was run on a subset of 208 spectra from this same dataset, also using ChemSpider candidates [6]. In CASMI2016, MetFrag alone ranked 11% (24 of 208) correct in first place, compared with 78% (162 of 208) using MetFrag, retention time, and reference information [6] (where reference information was the largest contributor to the improvement in ranking [5]). This shows that metadata is still very much needed for rapid prioritization in high-throughput tentative identification for well-known substances. However, as discussed above, reference information is not always applicable, and in these cases, HDX experiments can provide additional information for candidate selection and has the clear advantage of being based on experimental information.

As demonstrated in this study (and also by previous studies utilizing this approach), HDX improves compound identification by narrowing down the number of potential candidates based on both MS1 and MS/MS data. The application with an LC system fully flushed with deuterated solvent is considerably more expensive than normal LC-HRMS, in our case about 15 vs 0.30 Euros per run for the solvent. Considering the overall cost of running non-target screening and the associated data evaluation, which may amount to many 100s of Euros, this extra cost can be considered acceptable for the additional information gained, as long as the instrument time and sample volume is available for the additional runs. In many cases, it is complementary to the MS/MS or retention time information typically used. With the integration into MetFrag, a semi-automated evaluation of data from HDX experiments is possible, while in previous studies, the data had to be evaluated and interpreted manually.

The way the data processing was performed in this study took advantage of the fact that the substance identity was “known,” which was critical for the method development. The expected HDX species were predicted and the corresponding data could thus be extracted easily. In true untargeted experiments, the “undeuterated” precursor masses in MS1 must be matched to the “deuterated” precursor masses without knowledge of the correct structure up front. This can be achieved by looking for a mass difference of *X*×(2.014102–1.007825) = 1.006277(*X*) units within a given retention time window, which could be determined using experiments on known standards. The number of deuteriums, *X*, can then be deduced from the mass difference and used in MetFrag to rank the candidates. As demonstrated in Fig. 5, the deuterated substance retention times can shift slightly and—in some cases—quite dramatically. The results presented here indicate that large retention time shifts will not be expected for rather fast gradient separations typically used in screening methods. However, compounds eluting under isocratic conditions at low aqueous eluent fractions might be severely affected. Observations so far have occurred in a reproducible fashion over standard and sample measurements, such that some simple rules will help define appropriate retention time windows for these cases. Additional verification on different sample matrices and with further dual functionality standards would be needed to see exactly when the large retention time shifts are expected, for which substance classes and whether this effect varies in different sample matrices.

For a broader application to non-target screening, care must be taken that isotope peaks are not incorrectly assigned as potential deuterated masses in full scan data processing, as the mass difference between the ^13^C isotope peak of the undeuterated species and a potential monodeuterated species is 0.00292 Da, which is, e.g., 7 ppm difference at *m/z* 400. In terms of MS/MS acquisition, a narrow isolation window (~ 1 Da) is essential, such that isotope peaks are not present in the fragmentation spectrum to confuse interpretation. In terms of full scan data processing, this will require high-quality peak grouping to correctly assign isotope peaks to features (componentization), in both the normal and deuterated experiments. For cases that behave as expected (e.g., 100% of H exchanged for D as expected), this should be relatively straightforward, as the isotope peaks will also be shifted by 100%. However, for cases of incomplete exchange, things can rapidly become more complicated. If only partial exchange occurs (e.g., 30%), then the M+1 peaks will be a mixture of [M+D]^+^ and ^13^C-[M+H]^+^, which requires a resolution R = 35,000 at *m/z* = 100, R = 70,000 at *m/z* = 200, etc. to resolve the isotopologues. It would be possible to resolve these peaks up to approximately *m/z* = 400 (R = 140,000) using the Orbitrap instrument applied in these experiments, but not generally with a QTOF. For molecules with a large number of exchangeable hydrogens and high mass (e.g., glycosides with several sugars), complex spectra will be obtained, and a low level of “normal” hydrogen in the deuterium-flooded LC systems becomes relevant (e.g., at 99% deuterium purity and 40 labile hydrogens, the probability that all these 40 hydrogens are exchanged is only 66%). Similar issues would be observed using post-column HDX, as these also yield mixed spectra, rather than the very clean spectra observed here. It is possible to do back-calculations to account for this (as is routinely done in proteomics experiments, for instance), but adds complications to the data interpretation and is beyond the scope of the current article. Additionally, future studies will need to investigate additional adducts, the combination of positive and negative ionization results to extract the molecular ion, as well as incomplete exchange.

In this manuscript, we have made use of the CompTox Chemicals Dashboard as a host for lists of chemical structures, both undeuterated and HDX versions. Each of these lists required manual registration of the chemical structures (deuterated and undeuterated) into the underlying DSSTox database in order to be exposed via the Dashboard [26]. If the HDX approach proves to be of general value in analysis, the development of “HDX versions” of chemicals at registration may be possible, requiring the generation of deuterium-labeled forms of the chemicals to save as “related substances” by default. In many ways, this is similar to the generation of “MS-Ready” forms of the chemicals [40] that utilizes transformations of input chemicals to provide desalted, non-stereospecific forms to support mass spectrometry analyses. The generation of HDX forms of the chemicals could be done via the jar provided in the ESM or via the implementation of a set of transformation rules (e.g., D-exchange of OH, SH, NH, NH_2_, etc.) to provide the HDX-related substance to support this type of analysis. Alternatively, a “HDX download file” could be provided of the predicted HDX forms of the entire CompTox database, if external users would find this useful.

Due to the methodological and experimental efforts, it is considered unlikely that HDX experiments will be applied to NTS of environmental samples on a regular basis (in contrast to stable isotope labelling in certain metabolomics experiments); however, in special cases, it may offer crucial help in identification. These cases include the screening for toxicologically relevant compounds such as amines or phenols where HDX can be expected to provide detailed structural information, as demonstrated in this study.

## Electronic supplementary material

ESM 1 (PDF 3 MB)

ESM 2 (JAR 17236 kb)

ESM 3 (JAR 5970 kb)

ESM 4 (XLSX 755 kb)

ESM 5 (XLSX 36 kb)

## References

[1] Frainay C, Schymanski E, Neumann S, Merlet B, Salek R, Jourdan F, Yanes O (2018). Mind the gap: mapping mass spectral databases in genome-scale metabolic networks reveals poorly covered areas. Metabolites.

[2] Blaženović I, Kind T, Ji J, Fiehn O (2018). Software tools and approaches for compound identification of LC-MS/MS data in metabolomics. Metabolites.

[3] Freund DM, Hegeman AD (2017). Recent advances in stable isotope-enabled mass spectrometry-based plant metabolomics. Curr Opin Biotechnol.

[4] Mahieu NG, Patti GJ (2017). Systems-level annotation of a metabolomics data set reduces 25 000 features to fewer than 1000 unique metabolites. Anal Chem.

[5] Ruttkies C, Schymanski EL, Wolf S, Hollender J, Neumann S (2016). MetFrag relaunched: incorporating strategies beyond in silico fragmentation. J Cheminform.

[6] Schymanski EL, Ruttkies C, Krauss M, Brouard C, Kind T, Dührkop K, Allen F, Vaniya A, Verdegem D, Böcker S, Rousu J, Shen H, Tsugawa H, Sajed T, Fiehn O, Ghesquière B, Neumann S (2017). Critical assessment of small molecule identification 2016: automated methods. J Cheminform.

[7] Blaženović I, Kind T, Torbašinović H, Obrenović S, Mehta SS, Tsugawa H, Wermuth T, Schauer N, Jahn M, Biedendieck R, Jahn D, Fiehn O (2017). Comprehensive comparison of in silico MS/MS fragmentation tools of the CASMI contest: database boosting is needed to achieve 93% accuracy. J Cheminform.

[8] Lam W, Ramanathan R (2002). In electrospray ionization source hydrogen/deuterium exchange LC-MS and LC-MS/MS for characterization of metabolites. J Am Soc Mass Spectrom.

[9] Novak T, Helmy R, Santos I (2005). Liquid chromatography–mass spectrometry using the hydrogen/deuterium exchange reaction as a tool for impurity identification in pharmaceutical process development. J Chromatogr B.

[10] Muz M, Krauss M, Kutsarova S, Schulze T, Brack W (2017). Mutagenicity in surface waters: synergistic effects of carboline alkaloids and aromatic amines. Environ Sci Technol.

[11] Acter T, Kim D, Ahmed A, Ha J-H, Kim S (2017). Application of atmospheric pressure photoionization H/D-exchange mass spectrometry for speciation of sulfur-containing compounds. J Am Soc Mass Spectrom.

[12] Ohashi N, Furuuchi S, Yoshikawa M (1998). Usefulness of the hydrogen–deuterium exchange method in the study of drug metabolism using liquid chromatography-tandem mass spectrometry. J Pharm Biomed.

[13] Shah RP, Garg A, Putlur SP, Wagh S, Kumar V, Rao V, Singh S, Mandlekar S, Desikan S (2013). Practical and economical implementation of online H/D exchange in LC-MS. Anal Chem.

[14] Kostyukevich Y, Acter T, Zherebker A, Ahmed A, Kim S, Nikolaev E (2018). Hydrogen/deuterium exchange in mass spectrometry. Mass Spectrom Rev.

[15] Ahmed A, Kim S (2013). Atmospheric pressure photo ionization hydrogen/deuterium exchange mass spectrometry—a method to differentiate isomers by mass spectrometry. J Am Soc Mass Spectrom.

[16] Zherebker A, Kostyukevich Y, Kononikhin A, Roznyatovsky VA, Popov I, Grishin YK, Perminova IV, Nikolaev E (2016). High desolvation temperature facilitates the ESI-sourceH/D exchange at non-labile sites of hydroxybenzoic acids and aromatic amino acids. Analyst.

[17] Acter T, Cho Y, Kim S, Ahmed A, Kim B, Kim S (2015). Optimization and application of APCI hydrogen–deuterium exchange mass spectrometry (HDX MS) for the speciation of nitrogen compounds. J Am Soc Mass Spectrom.

[18] Strehmel N, Böttcher C, Schmidt S, Scheel D (2014). Profiling of secondary metabolites in root exudates of Arabidopsis thaliana. Phytochemistry.

[19] Ruttkies C, Strehmel N, Scheel D, Neumann S (2015). Annotation of metabolites from gas chromatography/atmospheric pressure chemical ionization tandem mass spectrometry data using an *in silico* generated compound database and MetFrag: annotation of metabolites from high-resolution GC/APCI-MS/MS data. Rapid Commun Mass Spectrom.

[20] Brack W, Altenburger R, Schüürmann G, Krauss M, López Herráez D, van Gils J, Slobodnik J, Munthe J, Gawlik BM, van Wezel A, Schriks M, Hollender J, Tollefsen KE, Mekenyan O, Dimitrov S, Bunke D, Cousins I, Posthuma L, van den Brink PJ, López de Alda M, Barceló D, Faust M, Kortenkamp A, Scrimshaw M, Ignatova S, Engelen G, Massmann G, Lemkine G, Teodorovic I, Walz K-H, Dulio V, Jonker MTO, Jäger F, Chipman K, Falciani F, Liska I, Rooke D, Zhang X, Hollert H, Vrana B, Hilscherova K, Kramer K, Neumann S, Hammerbacher R, Backhaus T, Mack J, Segner H, Escher B, de Aragão Umbuzeiro G (2015). The SOLUTIONS project: challenges and responses for present and future emerging pollutants in land and water resources management. Sci Total Environ.

[21] Hashmi MAK, Escher BI, Krauss M, Teodorovic I, Brack W (2018). Effect-directed analysis (EDA) of Danube River water sample receiving untreated municipal wastewater from Novi Sad, Serbia. Sci Total Environ.

[22] König M, Escher BI, Neale PA, Krauss M, Hilscherová K, Novák J, Teodorović I, Schulze T, Seidensticker S, Kamal Hashmi MA, Ahlheim J, Brack W (2017). Impact of untreated wastewater on a major European river evaluated with a combination of in vitro bioassays and chemical analysis. Environ Pollut.

[23] Schymanski EL, Singer HP, Longrée P, Loos M, Ruff M, Stravs MA, Ripollés Vidal C, Hollender J (2014). Strategies to characterize polar organic contamination in wastewater: exploring the capability of high resolution mass spectrometry. Environ Sci Technol.

[24] NORMAN Network NORMAN suspect list exchange. In: NORMAN Suspect List Exchange. https://www.norman-network.com/?q=node/236. Accessed 13 Mar 2019.

[25] US Environmental Protection Agency. EAWAGSURF: Eawag surfactants list: surfactants screened in Swiss wastewater 2014. 2019. https://comptox.epa.gov/dashboard/chemical_lists/EAWAGSURF. Accessed 13 Mar 2019.

[26] Williams AJ, Grulke CM, Edwards J, McEachran AD, Mansouri K, Baker NC, Patlewicz G, Shah I, Wambaugh JF, Judson RS, Richard AM (2017). The CompTox Chemistry Dashboard: a community data resource for environmental chemistry. J Cheminform.

[27] Mayfield J CDK Depict Web Interface. http://simolecule.com/cdkdepict/depict.html. Accessed 30 Oct 2018.

[28] Chambers MC, Maclean B, Burke R, Amodei D, Ruderman DL, Neumann S, Gatto L, Fischer B, Pratt B, Egertson J, Hoff K, Kessner D, Tasman N, Shulman N, Frewen B, Baker TA, Brusniak M-Y, Paulse C, Creasy D, Flashner L, Kani K, Moulding C, Seymour SL, Nuwaysir LM, Lefebvre B, Kuhlmann F, Roark J, Rainer P, Detlev S, Hemenway T, Huhmer A, Langridge J, Connolly B, Chadick T, Holly K, Eckels J, Deutsch EW, Moritz RL, Katz JE, Agus DB, MacCoss M, Tabb DL, Mallick P (2012). A cross-platform toolkit for mass spectrometry and proteomics. Nat Biotechnol.

[29] Stravs MA, Schymanski EL, Singer HP, Hollender J (2013). Automatic recalibration and processing of tandem mass spectra using formula annotation: recalibration and processing of MS/MS spectra. J Mass Spectrom.

[30] Willighagen EL, Mayfield JW, Alvarsson J, Berg A, Carlsson L, Jeliazkova N, Kuhn S, Pluskal T, Rojas-Chertó M, Spjuth O, Torrance G, Evelo CT, Guha R, Steinbeck C (2017). The Chemistry Development Kit (CDK) v2.0: atom typing, depiction, molecular formulas, and substructure searching. J Cheminform.

[31] Wohlgemuth G, Mehta SS, Mejia RF, Neumann S, Pedrosa D, Pluskal T, Schymanski EL, Willighagen EL, Wilson M, Wishart DS, Arita M, Dorrestein PC, Bandeira N, Wang M, Schulze T, Salek RM, Steinbeck C, Nainala VC, Mistrik R, Nishioka T, Fiehn O (2016). SPLASH, a hashed identifier for mass spectra. Nat Biotechnol.

[32] Steinbeck C, Han Y, Kuhn S, Horlacher O, Luttmann E, Willighagen E (2003). The Chemistry Development Kit (CDK): an open-source Java library for chemo- and bioinformatics. J Chem Inf Comput Sci.

[33] Steinbeck C, Hoppe C, Kuhn S, Floris M, Guha R, Willighagen E (2006). Recent developments of the Chemistry Development Kit (CDK)- an open-source Java library for chemo- and bioinformatics. Curr Pharm Des.

[34] Wolf S, Schmidt S, Müller-Hannemann M, Neumann S (2010). In silico fragmentation for computer assisted identification of metabolite mass spectra. BMC Bioinform.

[35] Kim S, Thiessen PA, Bolton EE, Chen J, Fu G, Gindulyte A, Han L, He J, He S, Shoemaker BA, Wang J, Yu B, Zhang J, Bryant SH (2016). PubChem substance and compound databases. Nucleic Acids Res.

[36] Pence HE, Williams A (2010). ChemSpider: an online chemical information resource. J Chem Educ.

[37] Reed DR, Kass SR (2001). Hydrogen—deuterium exchange at non-labile sites: a new reaction facet with broad implications for structural and dynamic determinations. J Am Soc Mass Spectrom.

[38] Kuck D (2007). Scrambling versus specific processes in gaseous organic ions during mass spectrometric fragmentation: elucidation of mechanistic origins by isotope labelling – an overview. J Label Compd Radiopharm.

[39] Schymanski EL, Jeon J, Gulde R, Fenner K, Ruff M, Singer HP, Hollender J (2014). Identifying small molecules via high resolution mass spectrometry: communicating confidence. Environ Sci Technol.

[40] McEachran AD, Mansouri K, Grulke C, Schymanski EL, Ruttkies C, Williams AJ (2018). “MS-Ready” structures for non-targeted high-resolution mass spectrometry screening studies. J Cheminform.

