# Supplementary material for: Supporting non-target identification by adding hydrogen deuterium exchange MS/MS capabilities to MetFrag
Source: Anal Bioanal Chem. 2019 Jun 17;411(19):4683–700. doi: 10.1007/s00216-019-01885-0 (PMC6611743; doi:10.1007/s00216-019-01885-0)
Supplement: Supplementary file 1 — (PDF 3 MB) [file 216_2019_1885_MOESM1_ESM.pdf]

Analytical and Bioanalytical Chemistry

Electronic Supplementary Material

Supporting non-target identification by adding hydrogen deuterium exchange MS/MS capabilities to MetFrag

Christoph Ruttkies, Emma L. Schymanski, Nadine Strehmel, Juliane Hollender, Steffen Neumann, Antony J. Williams, Martin Krauss

Additional files available under 10.1007/s00216-019-01885-0.

This file contains additional figures and tables (as indicated on the next page) to support the manuscript text. Further Electronic Supplementary Material (ESM) is provided containing two jar files (instructions see next page), as well as Tables S3 and S5 in excel format. Additionally, we note that the entire computational analysis can be downloaded and reproduced from the following URL: https://msbi.ipb-halle.de/download/metfrag-hdx/

convertSDF_readme.txt (Instruction to file ConvertSDF.jar)

```
http://www.rforrocks.de/wp-content/uploads/2012/10/ConvertSDF.jar

java -jar ConvertSDF.jar sdf='sdffile' out='output_folder' fast=true

the file has the same name like the sdf but ending csv
output_folder has to be defined

the resulting csv is pipe separated (|)
```

hdx_0.0.2_readME.txt (Instruction to file hdx-0.0.2.jar)

```
Link: https://msbi.ipb-halle.de/~cruttkie/metfrag/hdx-0.0.2.jar

It now should output the deuterated SMILES with stereo.


Simply run:

java -jar hdx-0.0.2.jar input.txt output.sdf

input.txt - should contain the SMILES strings one per line.

output.sdf - output SDF file with the properties:

####

MolecularFormula - mol. formula of native molecule

MonoisotopicMass - mono. mass of native molecule

ExchangedHydrogens - exchanged hydrogens

Identifier - line number of SMILES in input.txt

SMILES - SMILES of native molecule

dMonoisotopicMass - mono. mass of deuterated molecule

dSMILES - SMILES of deuterated molecule

dMolecularFormula - mono. mass of deuterated molecule

dMplusHplus - [M+H]+ mass of deuterated molecule/ion

dMplusDplus - [M+D]+ mass of deuterated molecule/ion

dMminusHminus - [M-H]- mass of deuterated molecule/ion

dMminusDminus - [M-D]- mass of deuterated molecule/ion
```

List of Tables

Table S1	*Summary of deuterated internal standards for method development*
Table S2	*Summary of compounds used in the second test set, plus rankings with H/D exchange information in positive mode*
Table S3	*Standard mix overview and acquisition summary – see additional ESM File*
Table S4	*Two fragment pairs from DEET and DEET-d7, exhibiting the expected (and observed) deuteration behaviour.*
Table S5	*Overview of Novi Sad sample results – see additional ESM File*

List of Figures

Figure S1	*DEET from MassBank (EA021313, HCD 90) compared with DEET-d7 (HCD 100), from measurements.*
Figure S2	*Top: Metolachlor (undeuterated spectrum) from MassBank (EA026813, HCD 90). Bottom: Metolachlor-d6 (HCD 100) as measured in this study.*
Figure S3	*Screenshots from Xcalibur (Thermo Scientific) showing the pesticide mix under normal chromatographic conditions (top) and HDX (bottom), in ESI positive mode.*
Figure S4	*Retention time (RT) under normal chromatographic conditions (x axis) versus delta RT (normal RT minus HDX retention time, y axis).*
Figure S5	*Screenshots from Xcalibur (Thermo Scientific) showing the Novi Sad dataset under normal chromatographic conditions (top) and HDX (bottom) for ESI positive mode.*
Figure S6	*Screenshots from Xcalibur (Thermo Scientific) showing the Novi Sad dataset under normal chromatographic conditions (top) and HDX (bottom) for ESI negative mode.*
Figure S7	*Chromatography of Metformin in the Novi Sad sample.*
Figure S8	*Metformin MS/MS spectra as observed in the standard mixes (to allow comparison with Figure 7 in the main text from the sample)*
Figure S9	*Chromatography (MS1) of dialkyl tetralin sulfonate (DATS) surfactants in the Novi Sad sample under normal (left) and HDX conditions (right).*
Figure S10	*Chromatography (MS1) of SPACs surfactants in the Novi Sad sample under normal (left) and HDX conditions (right).*

Extra Tables

Table S1 Summary of deuterated internal standards for method development

Details		Structure
Short Name:<br>ChemSpider ID (no D):<br>ChemSpider ID (with D):<br>DTXSID (no D)<br>DTXSID (with D)<br>MassBank IDs*:<br>CDK Depict Link:	DEET-d7<br>4133<br>D_7: 24533532<br>DTXSID2021995<br>DTXSID701010011<br>EA021311<br>DEET-d7	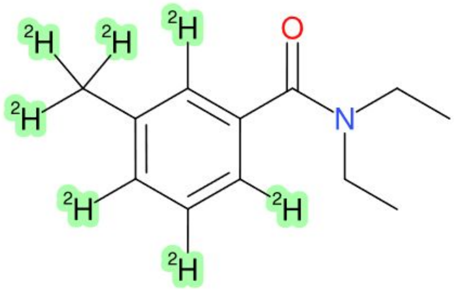
Short Name:<br>ChemSpider ID (no D):<br>ChemSpider ID (with D):<br>DTXSID (no D)<br>DTXSID (with D)<br>MassBank IDs*:<br>CDK Depict Link:	Metolachlor-d6<br>4025<br>D_6: 24532769<br>DTXSID4022448<br>DTXSID401010012<br>EA026806, EA026813<br>Metolachlor-d6	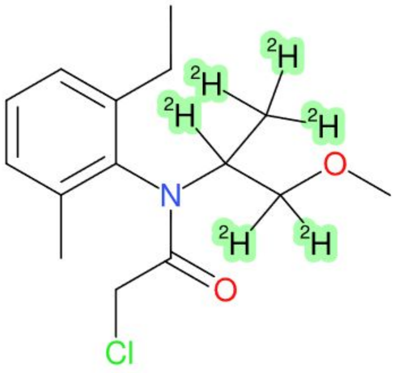
Short Name:<br>ChemSpider ID (no D):<br>ChemSpider ID (with D):<br>DTXSID (no D)<br>DTXSID (with D)<br>MassBank IDs*:<br>CDK Depict Link:	Carbamazepine-d10<br>2457<br>D_{10}: 24532296<br>DTXSID4022731<br>DTXSID30497060<br>EA019407<br>Carbamazepine-d10	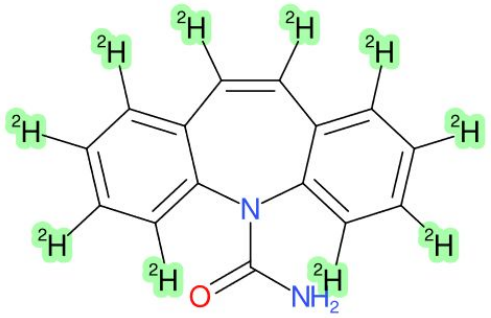

*most similar spectra, i.e. HCD90

Table S2 Summary of compounds used in the second test set, plus rankings with H/D exchange information in positive mode. The MetFrag results were ranked with different weights using Equation 6 and candidates from the PubChem database. The weights and rankings involving the deuterated terms are highlighted in blue. These initial results fed into the design of the main evaluation. $\omega_1 = \omega_{MetFrag}$, $\omega_2 = \omega_{MetFragHD}$, $\omega_3 = \omega_{PairHD}$ and $\omega_4 = \omega_{OSN}$ in Equation 6

Name,<br>PubChem ID,<br>DTXSID	Deuterated (HDX) Structure	ω_1	ω_2	ω_3	ω_4	Rank	Candidates
Kinetin<br>3830<br>DTXSID9035175<br><br>Kinetin-2d<br>DTXSID801010014<br>CDK Depict	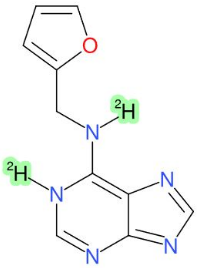	1.0	0.0	0.0	0.0	8	570
		0.4	0.4	0.1	0.1	6	
		0.3	0.3	0.2	0.2	7	
N-(3-Indolyl acetyl)-L-valine<br>446641<br>DTXSID70332229<br><br>N-(3-Indolyl acetyl)-L-valine-3d<br>DTXSID501010015<br>CDK Depict	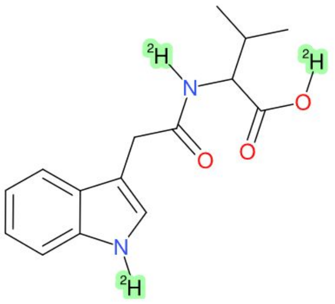	1.0	0.0	0.0	0.0	79	12709
		0.4	0.4	0.1	0.1	42	
		0.3	0.3	0.2	0.2	23	
o-Anisic acid<br>11370<br>DTXSID3060376<br><br>o-Anisic acid-1d<br>DTXSID201010016<br>CDK Depict	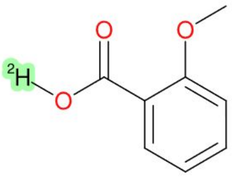	1.0	0.0	0.0	0.0	96	677
		0.4	0.4	0.1	0.1	45	
		0.3	0.3	0.2	0.2	45	
Phlorizin<br>6072<br>DTXSID3075339<br><br>Phlorizin-7d<br>DTXSID901010017<br>CDK Depict	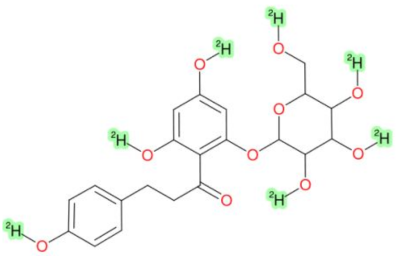	1.0	0.0	0.0	0.0	15	82
		0.4	0.4	0.1	0.1	6	
		0.3	0.3	0.2	0.2	4	

Table S3 This is an additional file containing details on the large standard mix, with the following tabs:

Table S3a	Mix acquisition summary
Table S3b	Mix isobar summary
Table S3c	Basic compound summary of all reference standards included (n=851)
Table S3d	Predicted HDX (using hdx-0.0.2 on SMILES_CompTox column of Table S3c, n=851)
Table S3e	Retention time summary of observed species, plus column and measurement. Note 4-hydroxybenzotriazole is listed twice as it was measured on both columns (n=593)
Table S3f	Chemical information for observed HDX species (n=593)
Table S3g	MS/MS Summary for observed species (n=593)

Table S4 Two fragment pairs from DEET and DEET-d7, exhibiting the expected (and observed) deuteration behaviour

Structure	*Fragment Pair 1*	*Fragment Pair 2*
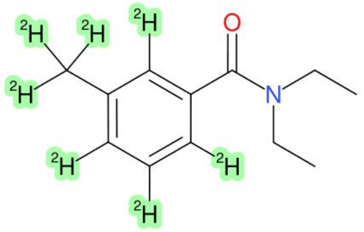	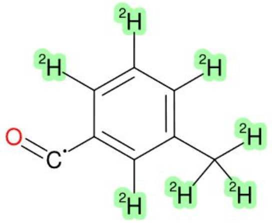	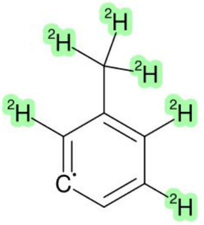
DEET-d7	*m/z = 126.0930 (C_8D_7O)*	*m/z = 97.0919 (C_7HD_6)*
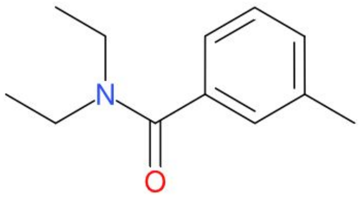	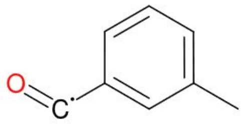	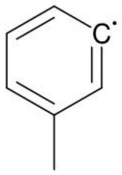
DEET	*m/z = 119.0492 (C_8H_7O)*	*m/z = 91.0543 (C_7H_7)*

Table S5 This is an additional file containing the environmental sample results

Table S5a	Target results with calculated deuterated species and observed pairs (n=107).
Table S5b	Summary of selected (tentatively identified) surfactant groups to demonstrate retention time shifts

Extra Figures

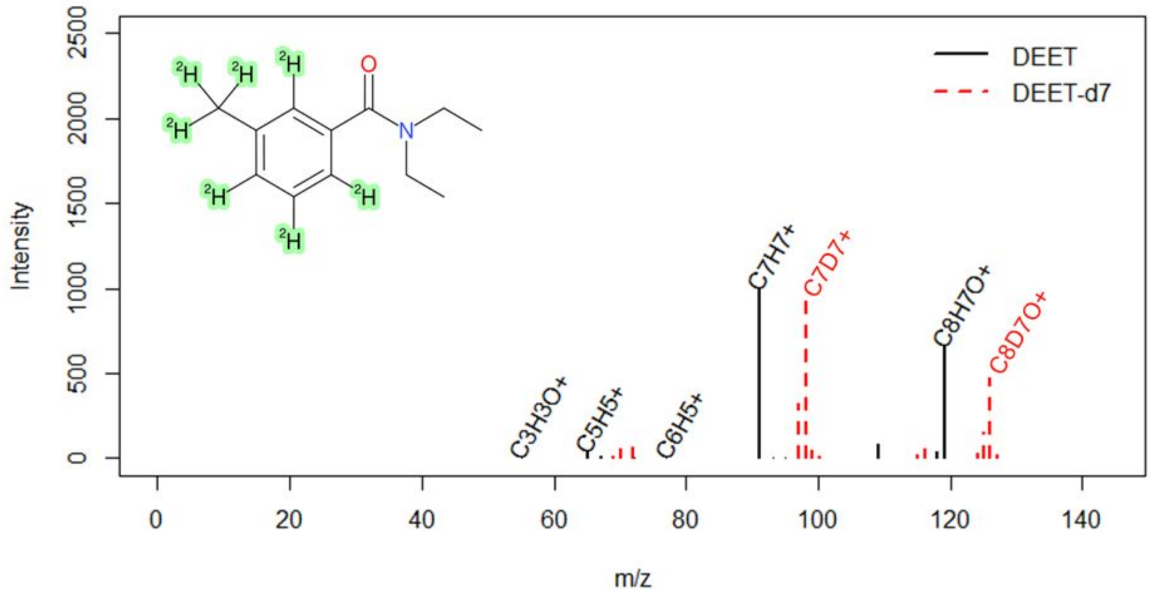

Fig. S1 DEET from MassBank (EA021313, HCD 90) compared with DEET-d7 (HCD 100), from measurements. The deuterated structure is shown in the inset

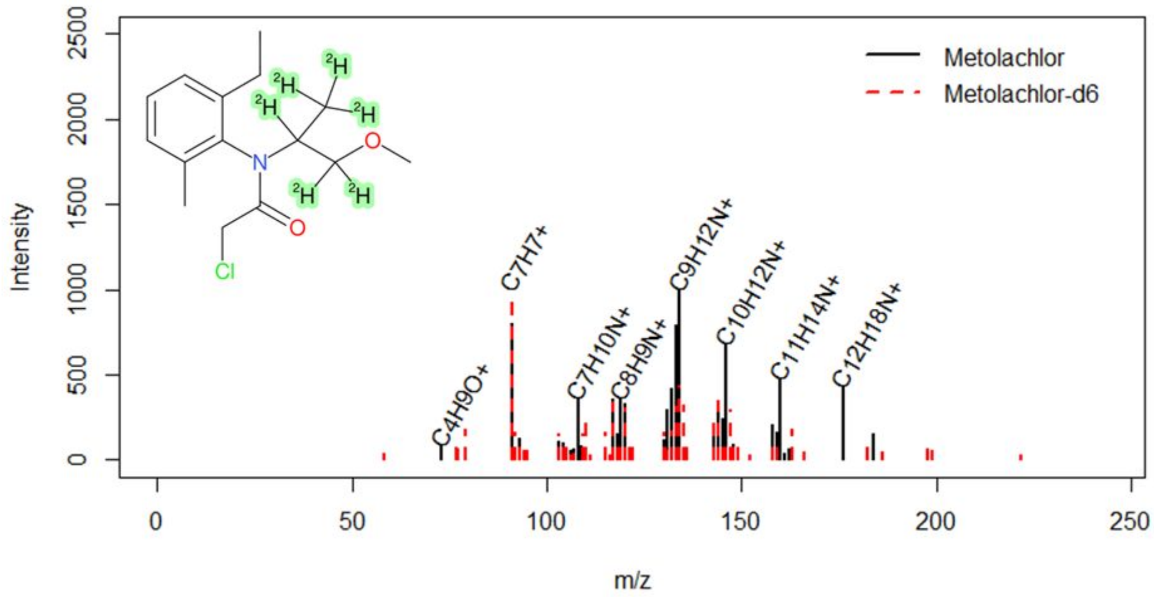

Fig. S2 Top: Metolachlor (undeuterated spectrum) from MassBank (EA026813, HCD 90). Bottom: Metolachlor-d6 (HCD 100) as measured in this study. The deuterated structure is shown in the inset

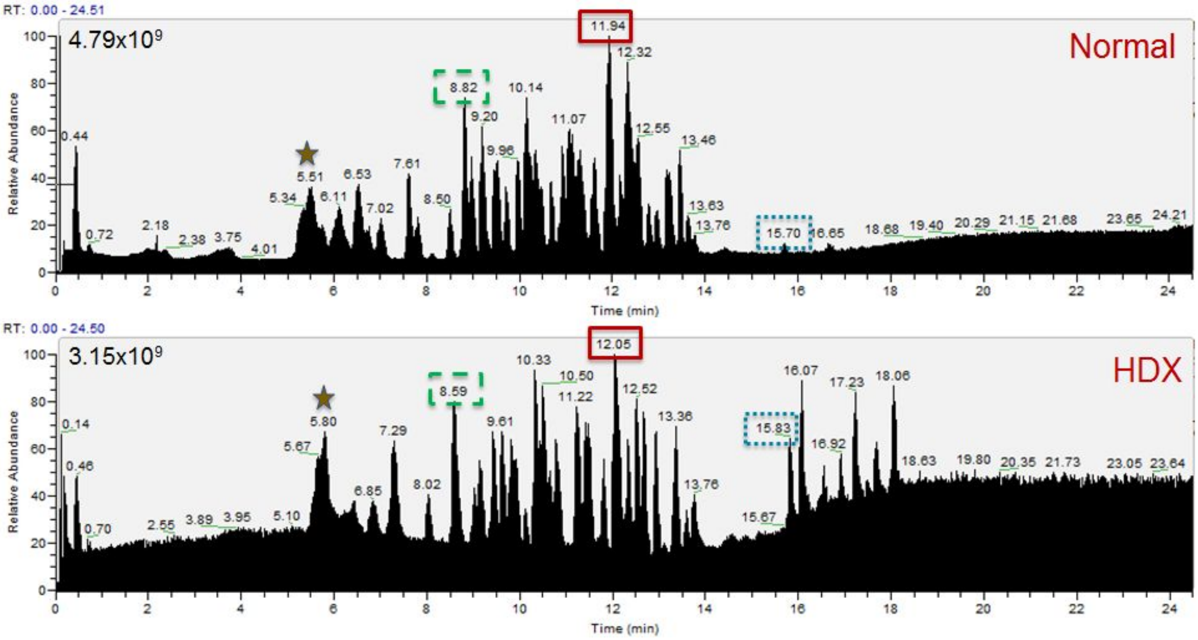

Fig. S3 Screenshots from Xcalibur (Thermo Scientific) showing the pesticide mix under normal chromatographic conditions (top) and HDX (bottom), both in ESI positive mode. The symbols (dark gold stars, green dashed box, red solid box and blue dotted box) indicate corresponding peak pairs; information about all substances is provided in Table S3

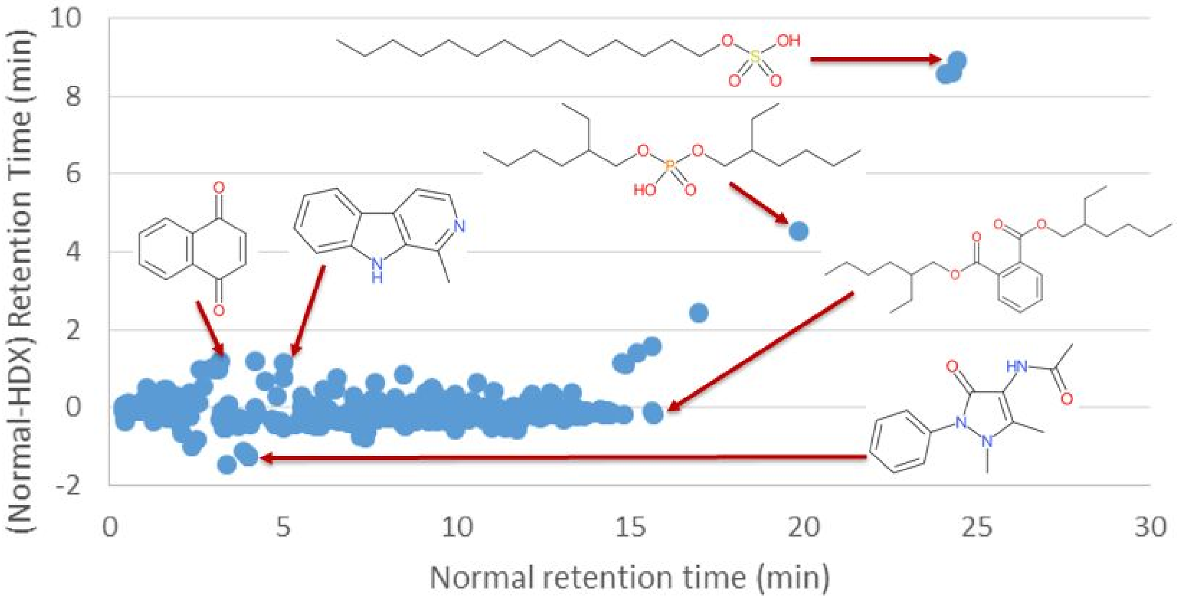

Fig. S4 Retention time (RT) under normal chromatographic conditions (x axis) versus delta RT (normal RT minus HDX retention time, y axis), along with representative structures of extreme data points. Full information on the retention times and substances are included in Table S3

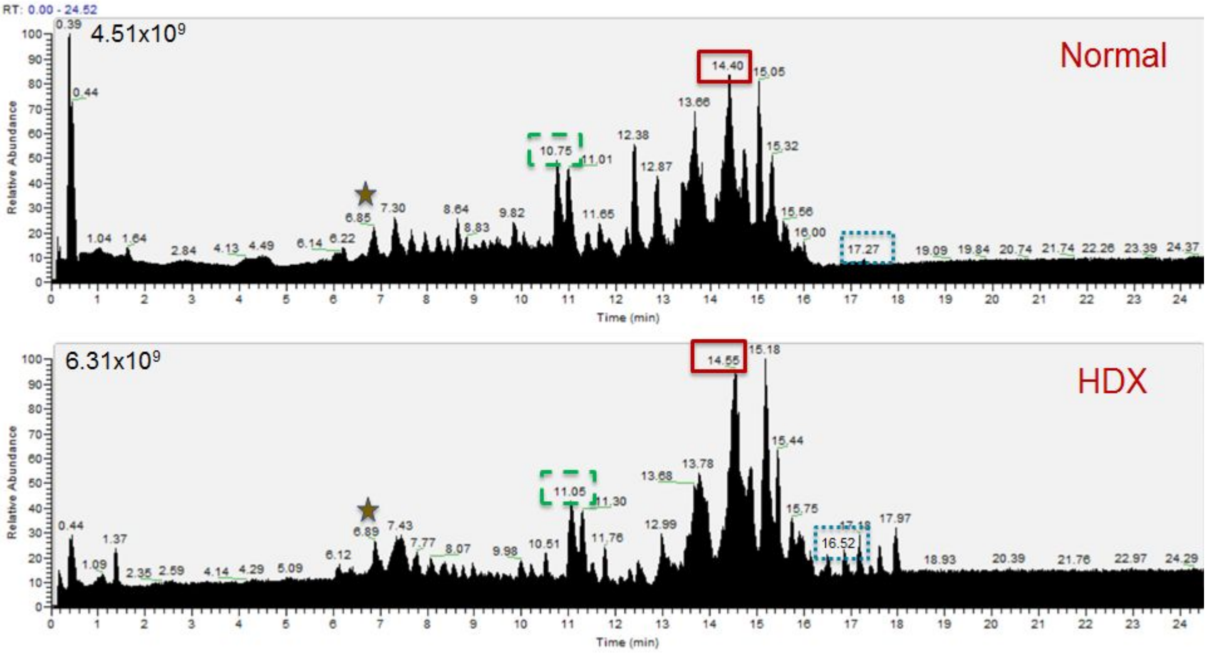

Fig. S5 Screenshots from Xcalibur (Thermo Scientific) showing the Novi Sad sample under normal chromatographic conditions (top) and HDX (bottom), both in ESI positive mode. The symbols (dark gold stars, green dashed box, red solid box and blue dotted box) indicate corresponding peak pairs; information about all substances is provided in Table S5

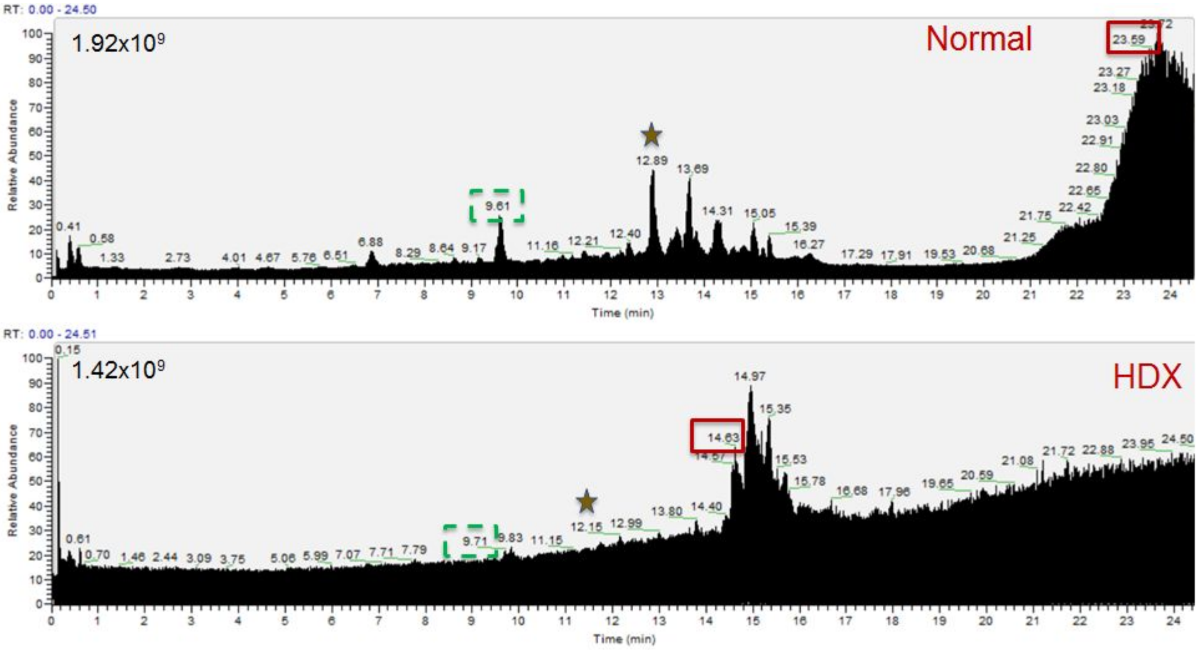

Fig. S6 Screenshots from Xcalibur (Thermo Scientific) showing the Novi Sad sample under normal chromatographic conditions (top) and HDX (bottom), both in ESI negative mode. The symbols (dark gold stars, green dashed box, red solid box) indicate corresponding peak pairs; information about all substances is provided in Table S5

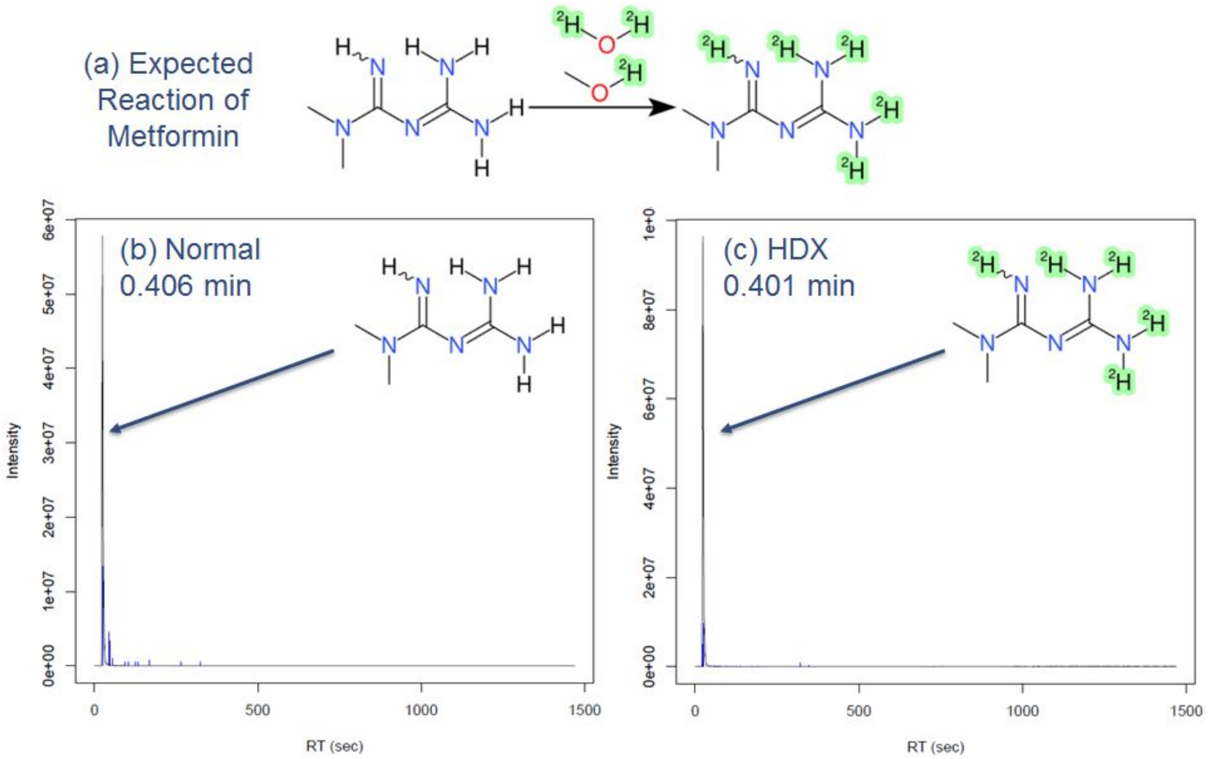

Fig. S7 Chromatography of Metformin in the Novi Sad sample (a) expected reaction, (b) normal chromatographic peak for $[M+H]^+$ and (c) observed $[M+D]^+$ peak in HDX measurements, with corresponding (neutral) species in the inset

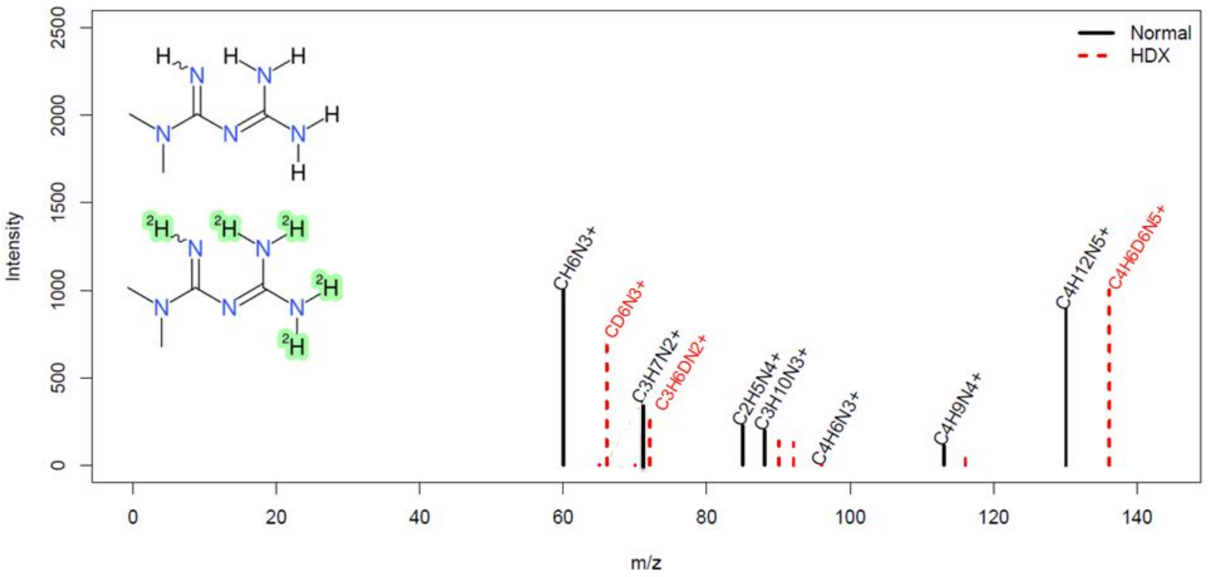

Fig. S8 Metformin MS/MS spectra as observed in the standard mixes (to allow comparison with Figure 7 in the main text from the sample)

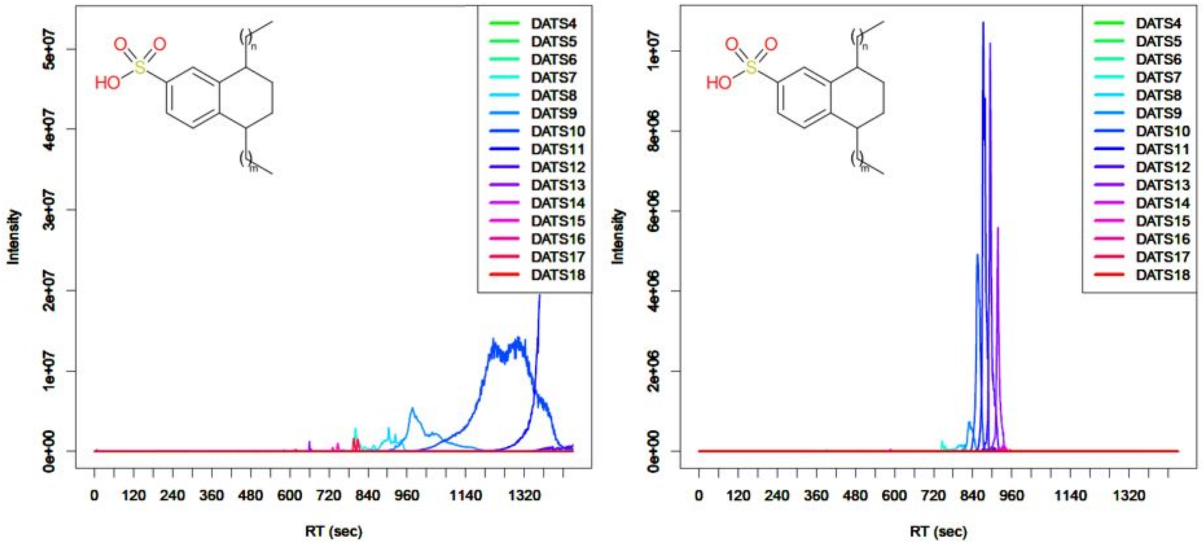

Fig. S9 Chromatography (MS1) of dialkyl tetralin sulfonate (DATS) surfactants (identification Level 3) in the Novi Sad sample under normal (left) and HDX conditions (right). This demonstrates that the sharpening of the peaks and associated large retention time shifts observed for these multifunctional structures in the mixtures were also observed in samples measured under the same conditions

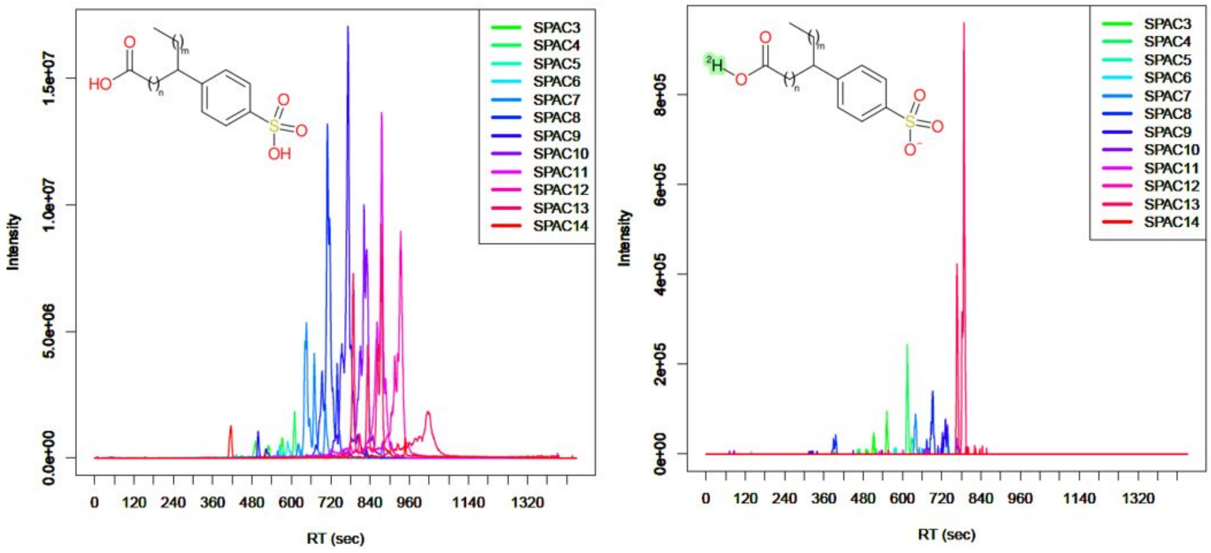

Fig. S10 Chromatography (MS1) of SPACs surfactants (identification Level 3) in the Novi Sad sample under normal (left) and HDX conditions (right). These peaks eluted earlier and did not appear to demonstrate large shifts, however there was insufficient intensity to form a clear picture of their behaviour or obtain MS/MS spectra that would have had a D incorporated in the fragments
